# Obesity causes mitochondrial fragmentation and dysfunction in white adipocytes due to RalA activation

**DOI:** 10.1038/s42255-024-00978-0

**Published:** 2024-01-29

**Authors:** Wenmin Xia, Preethi Veeragandham, Yu Cao, Yayun Xu, Torrey E. Rhyne, Jiaxin Qian, Chao-Wei Hung, Peng Zhao, Ying Jones, Hui Gao, Christopher Liddle, Ruth T. Yu, Michael Downes, Ronald M. Evans, Mikael Rydén, Martin Wabitsch, Zichen Wang, Hiroyuki Hakozaki, Johannes Schöneberg, Shannon M. Reilly, Jianfeng Huang, Alan R. Saltiel

**Affiliations:** 1 Division of Endocrinology and Metabolism, Department of Medicine, University of California San Diego, San Diego, CA USA; 2 Electron Microscopy Core, Cellular and Molecular Medicine, University of California San Diego, San Diego, CA USA; 3 Department of Biosciences and Nutrition, Karolinska Institute, Stockholm, Sweden; 4 Storr Liver Centre, Westmead Institute for Medical Research and Westmead Hospital, University of Sydney School of Medicine, Sydney, New South Wales Australia; 5 Gene Expression Laboratory, Salk Institute for Biological Studies, San Diego, CA USA; 6 Department of Medicine (H7), Karolinska Institute (C2-94), Karolinska University Hospital, Stockholm, Sweden; 7 Department of Pediatrics and Adolescent Medicine, Division of Pediatric Endocrinology and Diabetes, Ulm University Medical Center, Ulm, Germany; 8 Department of Pharmacology, University of California San Diego, San Diego, CA USA; 9 Department of Chemistry and Biochemistry, University of California San Diego, San Diego, CA USA; 10 Present Address: Department of Biochemistry and Structural Biology, University of Texas Health Science Center, San Antonio, TX USA; 11 Present Address: Weill Center for Metabolic Health, Division of Endocrinology, Diabetes and Metabolism, Department of Medicine, Weill Cornell Medicine, New York, NY USA

**Keywords:** Energy metabolism, Obesity, Mechanisms of disease, Metabolism, Mitochondria

## Abstract

Mitochondrial dysfunction is a characteristic trait of human and rodent obesity, insulin resistance and fatty liver disease. Here we show that high-fat diet (HFD) feeding causes mitochondrial fragmentation in inguinal white adipocytes from male mice, leading to reduced oxidative capacity by a process dependent on the small GTPase RalA. RalA expression and activity are increased in white adipocytes after HFD. Targeted deletion of RalA in white adipocytes prevents fragmentation of mitochondria and diminishes HFD-induced weight gain by increasing fatty acid oxidation. Mechanistically, RalA increases fission in adipocytes by reversing the inhibitory Ser637 phosphorylation of the fission protein Drp1, leading to more mitochondrial fragmentation. Adipose tissue expression of the human homolog of Drp1, *DNM1L*, is positively correlated with obesity and insulin resistance. Thus, chronic activation of RalA plays a key role in repressing energy expenditure in obese adipose tissue by shifting the balance of mitochondrial dynamics toward excessive fission, contributing to weight gain and metabolic dysfunction.

## Abstract (web summary)

Xia et al. show that the activity of the small GTPase RalA is increased in white adipocytes in diet-induced obese mice. RalA enhances mitochondrial fission and therefore reduces energy expenditure, which contributes to weight gain.

## Main

Obesity has become a worldwide epidemic^1^, dramatically increasing the incidence of type 2 diabetes, nonalcoholic steatohepatitis and other cardiometabolic abnormalities^2–4^. During the development of obesity, white adipose tissue (WAT) chronically expands and undergoes metabolic changes characterized by hormone insensitivity, inflammation, fibrosis and apoptosis^5,6^. While mitochondria play an important metabolic role in healthy adipocytes, oxidizing fuel to produce ATP and generating heat during thermogenesis, mitochondrial function is impaired in obese individuals^7–10^; however, what drives mitochondrial damage and how it contributes to obesity and its many complications remains unknown.

Obesity is associated with hyperinsulinemia and diabetes^11,12^ and studies have suggested a link between mitochondrial dysfunction, reduced energy expenditure and insulin resistance^13^. Altered mitochondrial oxidative function has been observed in muscle as well as adipose tissue from obese compared to healthy weight individuals^14–18^ and adipocytes from obese individuals contain fewer mitochondria compared to lean counterparts^15^. Moreover, the mitochondria in the muscle of obese individuals are fragmented^14^. Changes in mitochondrial size and number are controlled by the dynamic balance of fusion and fission^19^. Fusion is crucial for the optimal control of mitochondrial number and integrity, particularly in response to changes in energy needs. Fission, which is catalyzed by the dynamin-related protein Drp1, mediates mitochondrial division and quality control during cell division^20^; however, mitochondrial fusion and fission are observed in many nondividing cells, indicating that the correct balance of these processes is crucial to adapting to energy needs and ensuring homeostasis.

Ral GTPases are members of the Ras superfamily involved in multiple cellular processes. We previously demonstrated that RalA is activated by insulin in adipocytes and in turn interacts with members of the exocyst complex to target GLUT4 vesicles to the plasma membrane for docking and subsequent fusion, leading to increased glucose uptake^21–23^. Insulin activates RalA through inhibitory phosphorylation of the RalGAP complex^24^, as well as localization of RGL2, a guanine-nucleotide exchange factor (GEF) for RalA^25^. In vivo activation of RalA through targeted deletion of the RalGAP protein *Ralgapb* promotes glucose uptake into brown adipose tissue (BAT)^26^ and dramatically improves glucose homeostasis in mice on HFD. Similarly, targeted deletion of *Ralgapa1* in mice improves postprandial glucose and lipid disposal into muscle^27^.

We report here that RalA gene and protein expression and activity are increased in adipocytes from obese mice and further that targeted deletion of *Rala* in white, but not brown, adipocytes attenuates HFD-induced obesity, due to dramatically increased energy expenditure and mitochondrial oxidative phosphorylation, specifically in inguinal WAT (iWAT). These beneficial effects of RalA deletion were driven by a reversal of the increased mitochondrial fission in white adipocytes induced by feeding mice HFD. In vitro studies revealed that RalA interacts with the protein phosphatase PP2Aa to promote the dephosphorylation of inhibitory Serine637 on Drp1, rendering Drp1 active, leading to excessive fission and mitochondrial fragmentation. Taken together, these data reveal that persistent elevation of RalA in obesity produces mitochondrial dysfunction in white adipocytes, with profound effects on systemic metabolism.

## Results

### White adipocyte-specific *Rala* deletion protects mice from high-fat-diet-induced obesity

**Fig. 1:**
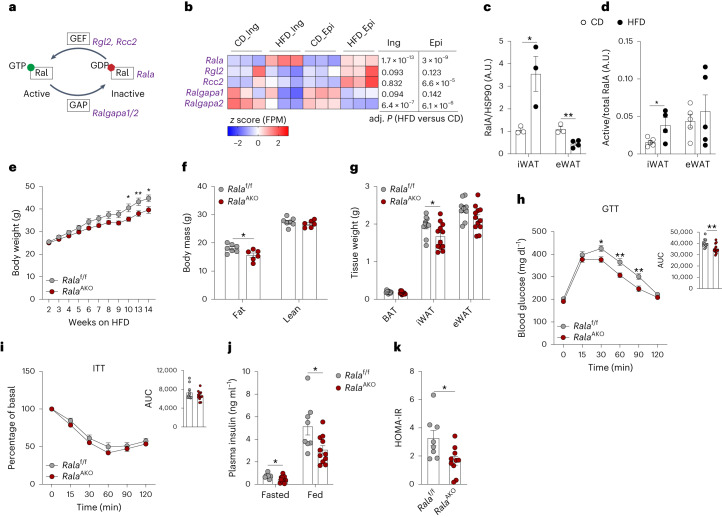
White adipocyte-specific *Rala* deletion protects mice from high-fat-diet-induced obesity. **a**, Scheme illustrating RalA activation network involving genes encoding RalA, GEF and GAP. **b**, RNA-seq analysis of primary inguinal (Ing) and epididymal (Epi) mature adipocytes isolated from mice (*n* = 3) under 16-week HFD feeding. Heat map displays transcriptional expression as *z*-scored FPM values. Adjusted *P* (adj. *P*) values are indicated and considered significant with values <0.05. **c**, Quantification of RalA protein content in mature adipocytes from iWAT and eWAT of mice fed with CD (*n* = 3) or HFD (*n* = 4) for 16 weeks. iWAT *P* = 0.033 CD versus HFD, eWAT *P* = 0.005 CD versus HFD. A.U., arbitrary units. **d**, Quantification of RalA GTPase activity in iWAT and eWAT of mice (*n* = 4) fed with CD or HFD for 4 weeks. iWAT *P* = 0.0448 CD versus HFD. **e**, Body weight of *Rala*^f/f^ (*n* = 8) and *Rala*^AKO^ (*n* = 10) mice fed with 60% HFD. Longitudinal graph, *P* = 0.0158, *P* = 0.009, *P* = 0.0106. **f**, Body mass of *Rala*^f/f^ (*n* = 7) and *Rala*^AKO^ (*n* = 6) mice fed with HFD for 12 weeks. Fat mass *P* = 0.0252. **g**, Fat depot weights of *Rala*^f/f^ (*n* = 10) and *Rala*^AKO^ (*n* = 12) mice fed with HFD for 12 weeks. iWAT *P* = 0.0465. **h**, GTT on 11-week HFD-fed *Rala*^f/f^ (*n* = 10) and *Rala*^AKO^ (*n* = 13) mice, *P* = 0.0174, *P* = 0.0036, *P* = 0.0069; the area under the curve (AUC) was calculated from longitudinal charts, *P* = 0.0062. **i**, ITT on 12-week HFD-fed *Rala*^f/f^ (*n* = 10) and *Rala*^AKO^ (*n* = 12) mice; AUC was calculated from longitudinal chart. **j**, Plasma insulin levels in 8-week HFD-fed *Rala*^f/f^ and *Rala*^AKO^ mice (*n* = 11). Fasted *P* = 0.0166. Fed *P* = 0.0329. **k**, HOMA-IR was calculated using fasting glucose and insulin levels from 8-week HFD-fed *Rala*^f/f^ (*n* = 8) and *Rala*^AKO^ (*n* = 10) mice. *P* = 0.0152. Data (**c**–**k**) show mean ± s.e.m., *P < 0.05, **P < 0.01, by two-tailed Student’s *t*-test (**c**,**d**,**f**,**g**,**j**,**k**) or two-way analysis of variance (ANOVA) with Bonferroni’s post-test (**e**,**h**,**i**). Source data

**Extended Data Fig. 1:**
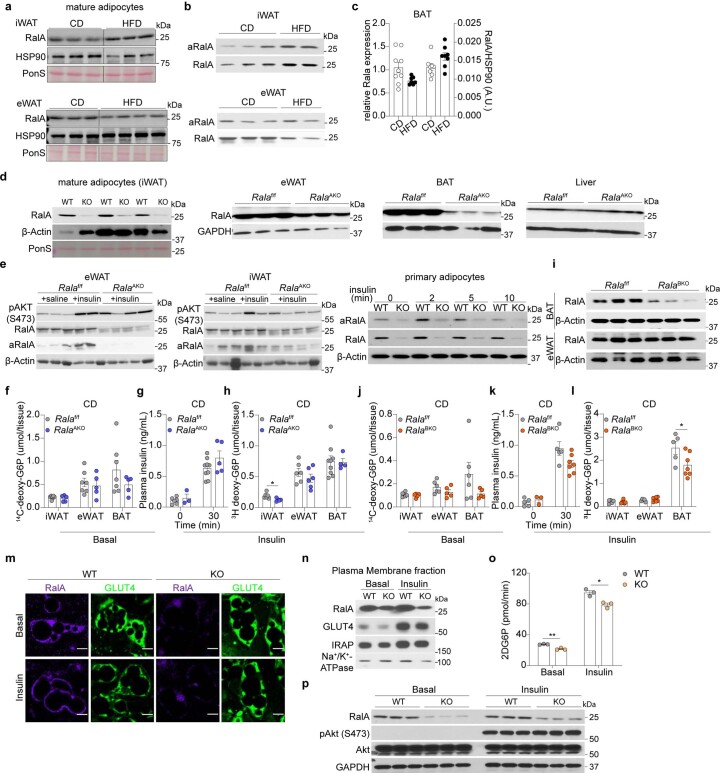
RalA protein content and activity are increased in obese adipocytes. **a**, Immunoblotting for RalA in mature adipocytes isolated from iWAT (n = 3) or eWAT (n = 4) of age-matched CD-fed (lean) mice and HFD-fed (obese) mice. **b**, Representative immunoblotting for active RalA (aRalA) in iWAT (upper panel) or eWAT (lower panel) of age-matched CD-fed mice (n = 3) and HFD-fed mice (n = 2). **c**, RalA mRNA expression and quantified protein levels in BAT of age-matched CD-fed and HFD-fed mice (n = 8). **d**, Immunoblotting of RalA in inguinal mature adipocyte fraction, eWAT, BAT, and liver from lean mice (n = 3). **e**, *In vivo* and *in vitro* activation of RalA by insulin in adipose tissue of *Rala*^f/f^ (saline n = 3, insulin n = 2) and *Rala*^AKO^ (n = 4) mice and primary adipocytes. 0.5U/kg or 100 nM insulin was injected or treated for 5 min or indicated time. **f**, Basal *in vivo* glucose uptake in 6 hrs fasted CD-fed mice injected with 10μCi [^14^C]-2deoxyglucose for 30 min (*Rala*^f/f^ n = 7, *Rala*^AKO^ n = 5). **g**, Plasma insulin levels before and 30 min after glucose injection (*Rala*^f/f^ n = 9, *Rala*^AKO^ n = 5). **h**, Insulin stimulated *in vivo* glucose uptake in CD-fed mice injected with 1.2 g/kg glucose and 10 μCi [^3^H]-2-deoxy-glucose for 30 min (*Rala*^f/f^ n = 6, *Rala*^AKO^ n = 9). **i**, Immunoblotting of RalA in BAT (upper panel) and eWAT (lower panel) of *Rala*^f/f^ and *Rala*^BKO^ mice (n = 3). **j**, Basal *in vivo* glucose uptake in 6 hrs fasted CD-fed mice injected with 10 μCi [^14^C]-2-deoxy-glucose for 30 min (*Rala*^f/f^ n = 7, *Rala*^BKO^ n = 5). **k**, Plasma insulin levels before and 30 min after glucose injection (*Rala*^f/f^ n = 5, *Rala*^BKO^ n = 7). **l**, Insulin stimulated *in vivo* glucose uptake in CD-fed mice injected with 1.2 g/kg glucose and 10 μCi [^3^H]-2-deoxy-glucose for 30 min (*Rala*^f/f^ n = 5, *Rala*^BKO^ n = 7). **m**, Representative immunostaining of endogenous RalA and GLUT4 in primary adipocytes treated with insulin (100 nM) or vehicle for 30 min, scale bar = 15 μm. (n = 3 biological samples). **n**, Representative immunoblotting of RalA, GLUT4, IRAP and Na + /K + -ATPase proteins in plasma membrane fraction of primary adipocytes treated with vehicle or insulin (100 nM) for 30 min. (n = 3 biological samples). **o**, 2-deoxy-glucose (2-DG) uptake in primary adipocytes treated with insulin (100 nM) or vehicle for 30 min (n = 3 biological samples). **p**, Immunoblotting of phosphor-Akt (S473), total Akt and GAPDH in primary adipocytes treated with or without insulin (100 nM) for 15 min. (n = 3 biological samples). The data are shown as the mean ± SEM, *P < 0.05, **P < 0.01 by two-tailed Student’s *T*-test (**h**, **l**, **o**). Source data

RNA sequencing (RNA-seq) analysis from isolated mature adipocytes derived from control and HFD-fed mice^28^ revealed that *Rala* expression is significantly upregulated in adipocytes from epididymal WAT (eWAT) and iWAT during obesity development, whereas *Ralgapa2* expression is downregulated (Fig. 1a,b). In addition, RalA protein content is increased in mature adipocytes from iWAT of obese mice (Fig. 1c and Extended Data Fig. 1a), accompanied by elevation of RalA–GTP binding (Fig. 1d and Extended Data Fig. 1b). These positive correlations seem to be exclusive for WAT as no changes in RalA levels were detected in BAT after HFD feeding (Extended Data Fig. 1c). Together, these observations support the notion that adipocyte RalA activity is constitutively elevated in obesity.

To explore further whether RalA plays a role in glucose homeostasis and energy metabolism, we generated adipocyte-specific *Rala* knockout (KO) (*Rala*^AKO^) mice by crossing *Rala*-floxed mice with adiponectin-Cre transgenic mice. Compared to *Rala*^f/f^ littermates, *Rala*^AKO^ mice had a greater than 90% decrease of RalA protein in primary adipocytes from WAT and BAT and an approximately 50% decrease in whole WAT, without changes in liver (Extended Data Fig. 1d). Insulin-stimulated GTP binding of RalA was diminished in WAT of *Rala*^AKO^ mice compared to control mice and reduced RalA activity was also observed in primary adipocytes (Extended Data Fig. 1e).

Depletion of RalA produced a reduction in insulin-stimulated glucose uptake in iWAT and BAT (Extended Data Fig. 1f–h). As our previous data showed that RalA plays an important role in regulating glucose uptake in BAT, we created brown adipocyte-specific KO (RalA^BKO^) mice by crossing RalA-floxed mice with UCP1-Cre transgenic mice (Extended Data Fig. 1i). In direct contrast to what was observed in brown adipocyte-specific RalGAP KO mice, glucose uptake was reduced in the BAT of RalA^BKO^ mice (Extended Data Fig. 1j–l). Notably, insulin-stimulated glucose uptake was mostly restricted to brown fat and we observed that RalA is dispensable for glucose uptake into eWAT in both gain-of-function and loss-of-function models. To examine further whether the impact of RalA on glucose uptake in adipocytes occurs in a cell-autonomous manner, we generated primary white adipocytes by differentiation of iWAT stromal vascular cells from control and KO mice. As previously seen in 3T3-L1 adipocytes^22^, KO of RalA completely prevented the translocation of GLUT4 from intracellular sites to the plasma membrane in response to insulin, as assessed by both microscopy and subcellular fractionation (Extended Data Fig. 1m,n). Moreover, insulin-stimulated glucose uptake in KO cells was significantly reduced in KO cells without disturbing upstream insulin signaling (Extended Data Fig. 1o,p).

**Extended Data Fig. 2:**
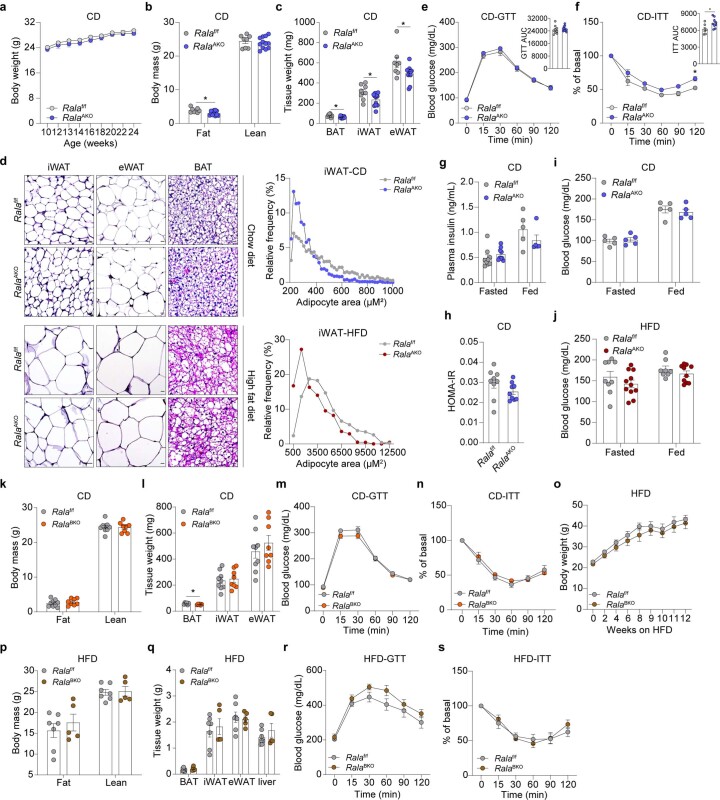
Brown adipocyte specific *Rala* deletion in mice did not phenocopy *Rala*^AKO^ mice. **a**-**c**, Body weight curve (**a**), body composition (**b**) and fat depot weights (**c**) of CD-fed *Rala*^f/f^ (n = 8) and *Rala*^AKO^ (n = 12) mice at the age of 24-weeks. *P* = 0.0103 (**b**). *P* = 0.0122, *P* = 0.0252, *P* = 0.0403 (**c**). **d**, Representative H&E staining images of iWAT, eWAT, and BAT from CD-fed and HFD-fed mice (n = 3), scale bar = 100 μm, representative adipocytes size quantification of iWAT from CD-fed and HFD-fed mice. **e**, **f**, Glucose tolerance test (GTT, **e**) and insulin tolerance test (ITT, **f**) on CD-fed *Rala*^f/f^ (n = 8) and *Rala*^AKO^ (n = 12) mice. Area under curves (AUC) were calculated from GTT and ITT, respectively. *P* = 0.0247 (**f**). **g**, Plasma insulin levels in CD-fed *Rala*^f/f^ and *Rala*^AKO^ mice under *ab libitum* (n = 5) or overnight fasted (n = 9) condition. **h**, Homeostasis model assessment-estimated insulin resistance (HOMA-IR) was calculated based on fasting glucose and insulin levels from CD-fed *Rala*^f/f^ and *Rala*^AKO^ mice (n = 9). **I**, Blood glucose levels in CD-fed mice (n = 5) at indicated states. **j**, Blood glucose levels in HFD-fed *Rala*^f/f^ (n = 9) and *Rala*^AKO^ (n = 11) mice at indicated states. **k**-**l**, Body composition (**k**) and fat depot weights (**l**) in CD-fed *Rala*^f/f^ (n = 9) and *Rala*^BKO^ (n = 8) mice at the age of 28 weeks. *P* = 0.0488 (**l**). **m**-**n**, GTT (**m**) and ITT (**n**) were performed in CD-fed *Rala*^f/f^ (n = 9) and *Rala*^BKO^ (n = 8) mice at the age of 26-weeks. **o**, Body weight curve of *Rala*^f/f^ (n = 6) and *Rala*^BKO^ (n = 4) mice fed with HFD for 12 weeks. **p**-**q**, Body composition (**p**) and fat depot and liver weights (**q**) in HFD-fed *Rala*^f/f^ (n = 7) and *Rala*^BKO^ (n = 5) mice at the age of 20-21 weeks. **r**, **s**, GTT (**r**) and ITT (**s**) were performed in HFD-fed *Rala*^f/f^ (n = 7) and *Rala*^BKO^ (n = 5) mice at the age of 17-weeks or 20-weeks, respectively. The data are presented as the mean ± SEM (**a**-**c**, **e**-**s**), *P < 0.05 by two-tailed Student’s *T*-test (**b**, **c**, **f**, **l**) or two-way ANOVA with Bonferroni’s post-test (**a**, **e**, **f**, **m-o**, **r**, **s**). Source data

Adipocyte-specific deletion of *Rala* had no effect on body weight in chow diet (CD)-fed mice, although these mice displayed a reduction in fat mass and depot weight (Extended Data Fig. 2a–c). Generally, adipocytes from iWAT were considerably smaller than those found in eWAT from mice fed CD^29^. *Rala*^AKO^ mice had smaller adipocytes in iWAT compared to control mice fed with CD, whereas adipocyte size was comparable in eWAT and BAT between the genotypes (Extended Data Fig. 2d). While *Rala*^AKO^ mice on CD showed no difference in glucose tolerance, there was a slight reduction in insulin tolerance when compared to *Rala*^f/f^ mice (Extended Data Fig. 2e,f). Insulin levels and homeostasis model assessment of insulin resistance (HOMA-IR) in *Rala*^AKO^ mice were not different from control mice fed with CD (Extended Data Fig. 2g,h); however, *Rala*^AKO^ mice gained significantly less weight than control littermates when challenged with 60% HFD (Fig. 1e), including a marked reduction of fat mass, with no change in lean body mass (Fig. 1f). Further analyses revealed that iWAT weight was reduced in *Rala*^AKO^ mice, with no difference in eWAT and BAT (Fig. 1g). HFD increased adipocyte size in all fat depots from wild-type (WT) mice, but the effect was most pronounced in iWAT; HFD-fed *Rala*^AKO^ mice displayed a trend toward smaller adipocytes in iWAT compared to control mice, but not in eWAT or BAT (Extended Data Fig. 2d). HFD-fed *Rala*^AKO^ mice exhibited a marked improvement in glucose tolerance compared to control mice, with no change in insulin tolerance (Fig. 1h,i), but with reduced insulin levels and improved HOMA-IR (Fig. 1j,k). Fasting glucose levels were comparable between the genotypes on either HFD or CD (Extended Data Fig. 2i,j).

To investigate further which adipose tissue depot is responsible for the reduced weight gain in *Rala*^AKO^ mice fed HFD, we placed *Rala*^BKO^ mice on HFD. Although CD-fed *Rala*^BKO^ mice showed a reduction in BAT weight, presumably due to reduced glucose uptake, there were no differences in overall fat mass or depot weight compared to control mice (Extended Data Fig. 2k, l). Glucose and insulin tolerance tests (GTTs and ITTs) were identical between the genotypes on control diet (Extended Data Fig. 2m,n). Moreover, no differences in body weight, fat mass, tissue weight, GTT or ITT were observed in HFD-fed *Rala*^BKO^ mice (Extended Data Fig. 2o–s). We note that HFD-fed mice exhibit insulin resistance in BAT, such that RalA activation is already decreased in WT mice on HFD compared to control diet. Thus, these results suggest that specific *Rala* deletion in WAT, especially in iWAT, protects mice against obesity.

### Loss of RalA in WAT ameliorates HFD-induced hepatic steatosis

**Fig. 2:**
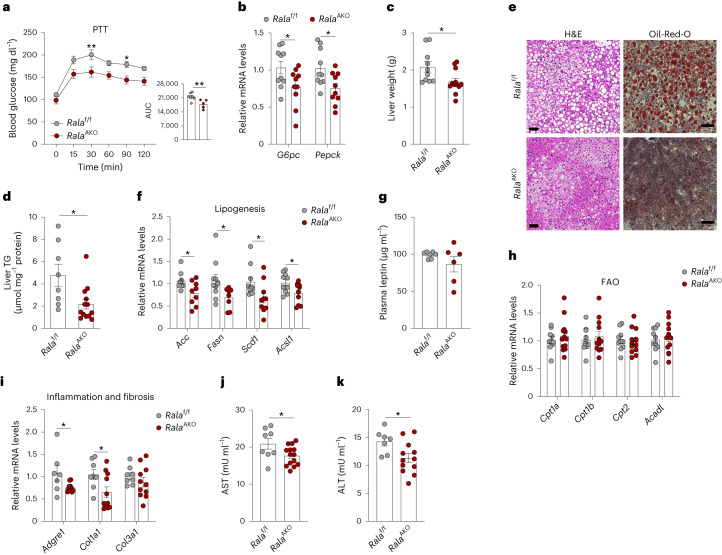
Loss of RalA in WAT ameliorates HFD-induced hepatic steatosis. **a**, A PTT was performed on overnight-fasted *Rala*^f/f^ (*n* = 7) and *Rala*^AKO^ (*n* = 5) mice after 8 weeks of HFD feeding; *P* = 0.0093, *P* = 0.0241. The AUC was calculated from a PTT longitudinal chart; *P* = 0.0097. **b**, Relative mRNA expression of key gluconeogenic genes in livers of HFD-fed *Rala*^f/f^ and *Rala*^AKO^ mice (*n* = 10). *P* = 0.0388, *P* = 0.0167. **c**, Liver weight of HFD-fed *Rala*^f/f^ (*n* = 10) and *Rala*^AKO^ (*n* = 12) mice; *P* = 0.0235. **d**, TG content in livers of HFD-fed *Rala*^f/f^ (*n* = 8) and *Rala*^AKO^ (*n* = 13) mice; *P* = 0.0129. **e**, Representative H&E staining image (left) and Oil-Red-O staining image (right) of liver sections in HFD-fed *Rala*^f/f^ and *Rala*^AKO^ mice (*n* = 3). Scale bar, 15 mm. **f**, Relative mRNA expression of lipogenic genes in livers of HFD-fed *Rala*^f/f^ (*n* = 9) and *Rala*^AKO^ (*n* = 10) mice; *P* = 0.0218, *P* = 0.0435, *P* = 0.0332, *P* = 0.0325. **g**, Plasma leptin levels in HFD-fed *Rala*^f/f^ (*n* = 7) and *Rala*^AKO^ (*n* = 6) mice. **h**, Relative mRNA expression of FAO-related genes in livers of HFD-fed *Rala*^f/f^ (*n* = 10) and *Rala*^AKO^ (*n* = 11) mice. **i**, Relative mRNA expression of genes related to inflammation and fibrosis in livers of HFD-fed *Rala*^f/f^ (*n* = 7) and *Rala*^AKO^ (*n* = 11) mice; *P* = 0.0347, *P* = 0.0325. **j**,**k**, Plasma AST (**j**) and ALT (**k**) activities in HFD-fed *Rala*^f/f^ (*n* = 7) and *Rala*^AKO^ (*n* = 14) mice; *P* = 0.0367(**j**), *P* = 0.0275 (**k**). Data (**a**–**d**,**f**–**k**) show mean ± s.e.m., *P < 0.05, **P < 0.01 by two-tailed Student’s *t*-test (**b**–**d**,**f**,**i**–**k**) or two-way ANOVA with Bonferroni’s post-test (**a**). Source data

As HFD-fed *Rala*^AKO^ mice showed an improved GTT without altering insulin tolerance, we speculated that the improved glucose handling is due to reduced hepatic glucose production. To test this assumption, we performed a pyruvate tolerance test (PTT) in HFD-fed *Rala*^f/f^ and *Rala*^AKO^ mice. *Rala*^AKO^ mice exhibited substantially lower glucose excursions following pyruvate challenge compared to control mice (Fig. 2a). There was a significant downregulation of the hepatic gluconeogenic genes *G6pc* and *Pepck* (Fig. 2b). These data suggest that adipocyte-specific *Rala* deletion improved glucose homeostasis partially through reduced hepatic glucose production.

Liver weights and triglyceride (TG) content were significantly reduced in HFD-fed *Rala*^AKO^ mice compared to control mice (Fig. 2c,d). Both hematoxylin and eosin (H&E) and Oil-Red-O staining indicated less lipid accumulation in the livers of *Rala*^AKO^ mice (Fig. 2e). In line with histology results, lipogenic genes (*Acc*, *Fasn*, *Scd1* and *Acsl1*) were expressed at lower levels in the livers of *Rala*^AKO^ mice (Fig. 2f); however, plasma leptin levels (Fig. 2g) and hepatic expression of genes related to fatty acid oxidation (FAO) (Fig. 2h) were unchanged in *Rala*^AKO^ mice. In addition, inflammatory (*Adgre1*) and fibrosis-related (*Col1a1* and *Col3a1*) genes were expressed at lower levels in livers of *Rala*^AKO^ mice (Fig. 2i), as were aspartate aminotransferase (AST) and alanine aminotransferase (ALT) activities (Fig. 2j,k). Of note, we did not observe a difference in liver weights in *Rala*^BKO^ compared to controls fed with HFD (Extended Data Fig. 2q). Together, these observations suggest that WAT-specific deletion of *Rala* systemically regulates lipid metabolism to ameliorate liver steatosis and damage in obesity.

### RalA deficiency in WAT increases energy expenditure and mitochondrial oxidative phosphorylation

**Fig. 3:**
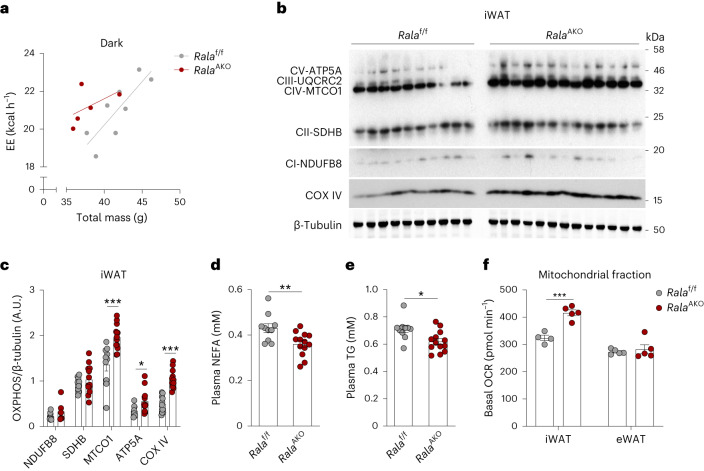
RalA deficiency in WAT increases energy expenditure and mitochondrial oxidative phosphorylation. **a**, Regression plot of energy expenditure (EE) measured in HFD-fed *Rala*^f/f^ (*n* = 8) and *Rala*^AKO^ (*n* = 5) mice during dark phase. ANCOVA was performed using body weight (BW) as a covariate, group effect *P* = 0.0391. **b**,**c**, Immunoblot (**b**) and quantification (**c**) of OXPHOS complex proteins and β-tubulin in iWAT of HFD-fed *Rala*^f/f^ (*n* = 10) and *Rala*^AKO^ (*n* = 13) mice. *P* = 0.0005, *P* = 0.0348, *P* < 0.0001. **d**,**e**, Plasma non-esterified fatty acid (NEFA; **d**) and TG (**e**) levels in HFD-fed *Rala*^f/f^ (*n* = 10) and *Rala*^AKO^ (*n* = 13) mice; *P* = 0.0077(**d**), *P* = 0.0115 (**e**). **f**, Basal OCR in mitochondria measured by Seahorse. Mitochondrial fractions were isolated from primary mature adipocytes in iWAT or eWAT of HFD-fed *Rala*^f/f^ (*n* = 4) and *Rala*^AKO^ (*n* = 5) mice. iWAT *P* = 0.0004. Data (**c**–**f**) show mean ± s.e.m., *P < 0.05, **P < 0.01, ***P < 0.001 by two-tailed Student’s *t*-test (**c**–**f**). Source data

**Extended Data Fig. 3:**
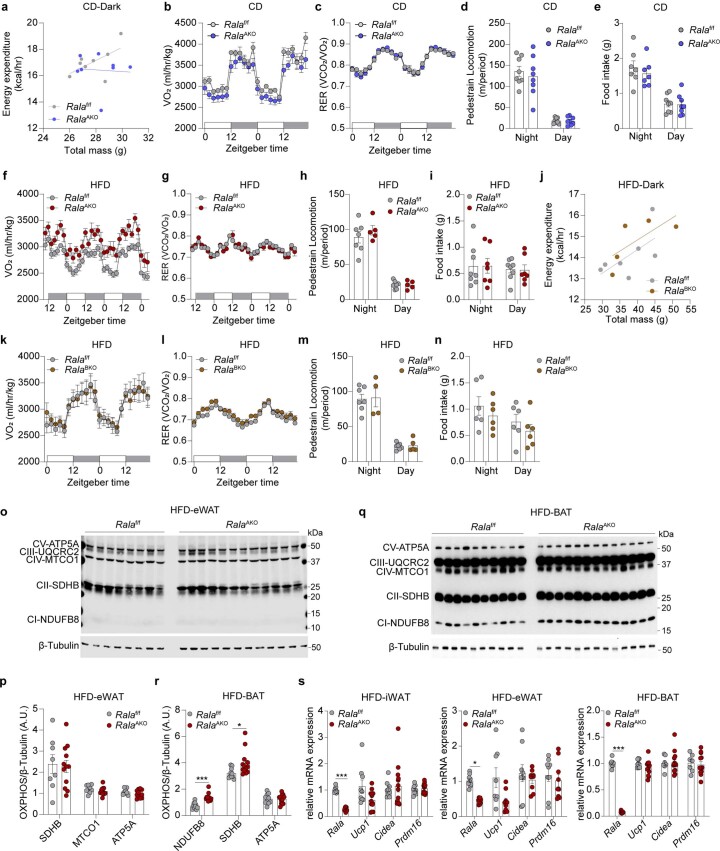
Neither CD-fed *Rala*^AKO^ mice nor HFD-fed *Rala*^BKO^ mice show increased energy expenditure. **a**, Regression plot of energy expenditure (EE) during dark phase against body weight (BW) in CD-fed *Rala*^f/f^ and *Rala*^AKO^ mice (n = 8). ANCOVA test using BW as a covariate, group effect *P* = 0.2805. **b**-**e**, BW-normalized oxygen consumption (**b**), respiratory exchange ratio (RER) (**c**), pedestrian locomotion (**d**) and food intake (**e**) over a two-days period were measured in CD-fed *Rala*^f/f^ and *Rala*^AKO^ mice (n = 8) by metabolic cages. **f**-**i**, BW-normalized oxygen consumption (**f**), respiratory exchange ratio (RER) (**g**), pedestrian locomotion (**h**) and food intake (**i**) over a two-days period were measured in HFD-fed *Rala*^f/f^ (n = 8) and *Rala*^AKO^ (n = 5) mice by metabolic cages. **j**, Regression plot of EE during dark phase against BW in HFD-fed *Rala*^f/f^ (n = 7) and *Rala*^BKO^ (n = 5) mice. ANCOVA test using BW as a covariate, group effect *P* = 0.2792. **k**-**n**, BW-normalized oxygen consumption (**k**), respiratory exchange ratio (RER, **l**), pedestrian locomotion (**m**) and food intake (**n**) over a two-days period were measured in HFD-fed *Rala*^f/f^ (n = 7) and *Rala*^BKO^ (n = 5) mice by metabolic cages. (**o**, **p**) Immunoblotting (**o**) and quantification (**p**) of OXPHOS proteins in eWAT of HFD-fed mice (*Rala*^f/f^ n = 8 and *Rala*^AKO^ n = 12). (**q**, **r**) Immunoblotting (**q**) and quantitation (**r**) of OXPHOS proteins in BAT of HFD-fed mice (*Rala*^f/f^ n = 10 and *Rala*^AKO^ n = 13). *P* < 0.0001, *P* = 0.0252 (**r**). **s**, Relative mRNA expression of browning-related genes in iWAT, eWAT and BAT of HFD-fed mice (*Rala*^f/f^ n = 8 and *Rala*^AKO^ n = 13). *P* < 0.0001, *P* < 0.0001, *P* < 0.0001. The data (**b**-**i**, **k**-**n**, **p**-**s**) are shown as the mean ± SEM, *P < 0.05, ***P < 0.001 by two-tailed Student’s *T*-tesI (**d**, **e**, **h**, **i**, **m**, **n**, **p**-**s**), or two-way ANOVA with Bonferroni’s post-test (**b**, **c**, **f**, **g**, **k**, **l**). Source data

To explore why adipose tissue *Rala* deletion protects mice from HFD-induced hepatic steatosis, weight gain and glucose intolerance, we investigated energy metabolism in *Rala*^AKO^ mice with metabolic cage studies. While *Rala* ablation in adipocytes did not affect energy metabolism and food intake in mice fed CD (Extended Data Fig. 3a–e), HFD-fed *Rala*^AKO^ mice displayed an increase in energy expenditure during the dark phase as determined by analysis of covariance (ANCOVA) using body weight as a covariate (Fig. 3a). Concordantly, oxygen consumption in *Rala*^AKO^ mice was similarly increased compared to controls (Extended Data Fig. 3f), although there was no difference in respiratory exchange rate (RER), locomotor activity or food intake between the genotypes (Extended Data Fig. 3g–i). In contrast, *Rala*^BKO^ mice fed HFD were identical to control littermates in energy expenditure, O_2_ consumption, RER, locomotor activity and food intake (Extended Data Fig. 3j–n). These observations demonstrate that *Rala* deficiency specifically in WAT increases energy expenditure.

Increased energy expenditure is an indirect reflection of increased mitochondrial oxidative activity^30^. Thus, we assessed the expression of mitochondrial proteins in fat depots. Oxidative phosphorylation (OXPHOS) proteins were markedly increased in iWAT of *Rala*^AKO^ mice (Fig. 3b,c), but not in eWAT (Extended Data Fig. 3o,p). Complex I and complex II levels were modestly increased in BAT of *Rala*^AKO^ mice (Extended Data Fig. 3q,r). This may occur because of systemic metabolic improvement in *Rala*^AKO^ mice rather than a cell-autonomous BAT function as HFD-fed *Rala*^BKO^ mice did not show an improved metabolic phenotype. In this regard, plasma free fatty acid (FFA) and TG levels in HFD-fed *Rala*^AKO^ mice were lower (Fig. 3d,e). Recent studies have shown that the beiging of iWAT promotes energy expenditure and protects against diet-induced obesity^31^. To test the possible involvement of a generalized browning of iWAT, we also examined thermogenic markers. *Ucp1*, *Cidea* and *Prdm16* expression was identical between the genotypes in all three fat depots, indicating that the improvement in energy expenditure in *Rala*^AKO^ mice did not reflect the development of beige adipose tissue (Extended Data Fig. 3s).

### *Rala* knockout in white adipocytes increases mitochondrial activity and fatty acid oxidation

**Fig. 4:**
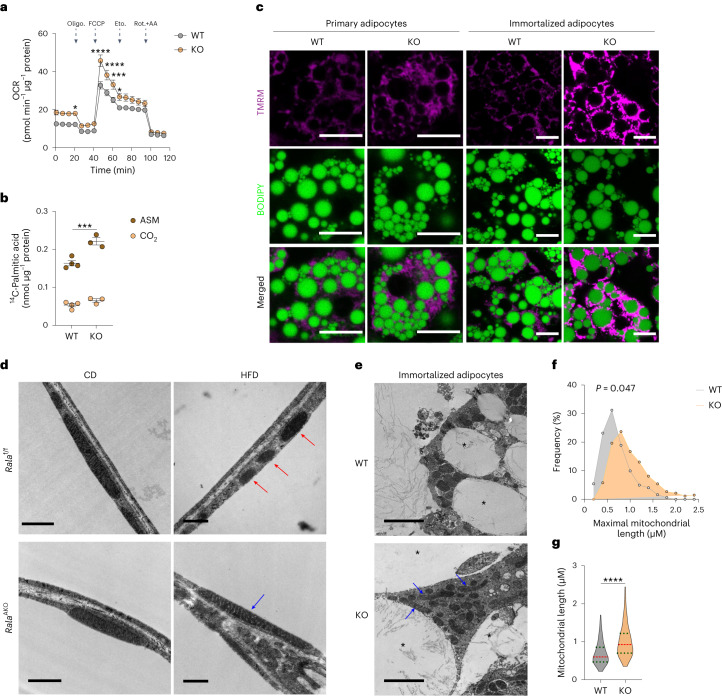
*Rala* knockout in white adipocytes increases mitochondrial activity and fatty acid oxidation via preventing obesity-induced mitochondrial fission in iWAT. **a**, OCR was measured in fully differentiated primary adipocytes (*n* = 8 biological samples); *P* = 0.0499, *P* < 0.0001, *P* < 0.0001, *P* = 0.0006, *P* = 0.0468. Vertical arrows indicate injection ports of indicated chemicals. **b**, ^14^C-PA oxidation in differentiated primary WT (*n* = 4 biological samples) and KO (*n* = 3 biological samples) adipocytes under basal conditions; *P* = 0.0037. **c**, Representative confocal images of live primary and immortalized adipocytes stained with TMRM (red) and BODIPY (green) (*n* = 3 biological samples). Scale bar, 15 μm. **d**, Representative transmission electron microscope (TEM) images of iWAT from CD-fed and HFD-fed *Rala*^f/f^ and *Rala*^AKO^ mice (*n* = 3 biological samples). Red arrow indicates fissed mitochondria; blue arrow indicates elongated mitochondria. Scale bar, 1 μm (CD) or 500 nm (HFD). **e**, Representative TEM images of WT and *Rala* KO immortalized adipocytes (*n* = 3 biological samples). Blue arrow indicates elongated mitochondria; asterisk indicates lipid droplet. Scale bar, 2 μm. **f**,**g**, Histogram (**f**) and violin plot (**g**) of maximal mitochondrial length in immortalized adipocytes (WT, six independent cells; KO, ten independent cells). Violin plot is presented as violin showing 25th to 75th percentiles and whiskers showing min to max; *P* = 0.047 (**f**), *P* < 0.0001 (**g**). Data (**a**,**b**) show mean ± s.e.m., *P < 0.05, ***P < 0.001, ****P < 0.0001 by two-tailed Student’s *t*-test (**b**,**g**), two-way ANOVA alone (**f**) or with Bonferroni’s post-test (**a**). Source data

**Extended Data Fig. 4:**
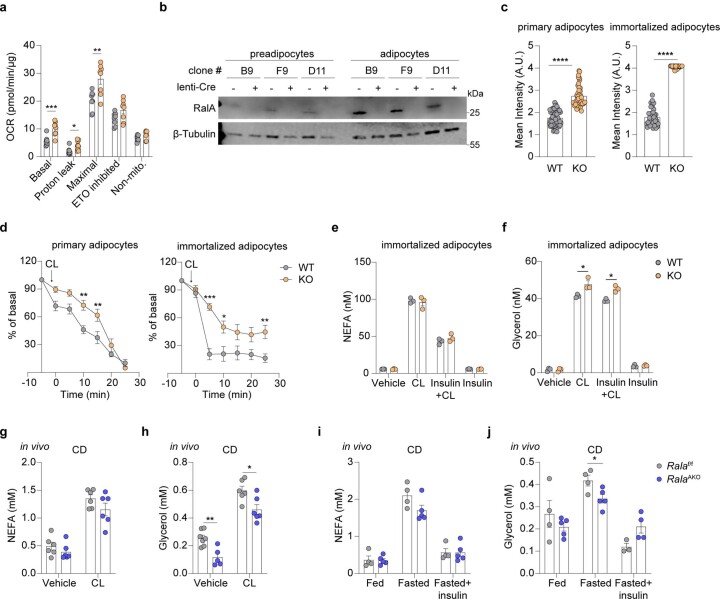
Absence of RalA in adipocytes did not affect free fatty acid release. **a**, Calculation of individual OCR in differentiated primary adipocytes (n = 8 biological samples). *P* = 0.0008, *P* = 0.0175, *P* = 0.0042. **b**, Representative immunoblot of RalA and β-Tubulin in immortalized preadipocytes and differentiated adipocytes. (n = 3 biological samples). **c**, Quantification of mean TMRM fluorescence intensity in primary adipocytes and immortalized adipocytes (n = 3 independent cells). *P* < 0.0001, *P* < 0.0001. **d**, Time course TMRM intensity quantification in primary and immortalized adipocytes (n = 4 biological samples). Adipocytes were treated with 1 μM CL-316,243 (CL) for indicated times. *P* = 0.0015, *P* = 0.0044 (left panel). *P* < 0.0001, *P* = 0.0154, *P* = 0.0095 (right panel). **e**, **f**, Quantification of NEFA (**e**) and free glycerol (**f**) released into medium from immortalized adipocytes (n = 3 biological samples). Cells were treated with 1 µM CL, 100 nM insulin, or in combination prior to medium collection. *P* = 0.0301, *P* = 0.0051 (**f**). **g**, **h**, Plasma levels of NEFA (**g**) and free glycerol (**h**) in CD-fed *Rala*^f/f^ and *Rala*^AKO^ mice (n = 6). *P* = 0.0027, *P* = 0.0173 (**h**). Mice were *i.p*. injected with CL or vehicle prior to blood sampling. **i**, **j**, Plasma levels of NEFA (**i**) and free glycerol (**j**) in CD-fed *Rala*^f/f^ (n = 4) and *Rala*^AKO^ (n = 5) mice. Mice were either subjected to *ad libitum* feeding, overnight fasting or fasting plus insulin injection 15 min prior to harvesting. *P* = 0.0316 (**j**). The data (**a**, **c**-**j**) are shown as the mean ± SEM, *P < 0.05, **P < 0.01, ***P < 0.001, ****P < 0.0001 by two-tailed Student’s *T*-test (**a**, **c**, **e**-**j**) or two-way ANOVA with Bonferroni’s post-test (**d**). Source data

We sought to evaluate further the mechanisms underlying improved energy metabolism in *Rala*^AKO^ mice and directly assessed mitochondrial activity in adipocytes. Measurements of basal respiration revealed that oxygen consumption rate (OCR) was increased in mitochondria isolated from KO iWAT compared to that from control mice, but was similar in eWAT mitochondria of *Rala*^f/f^ and *Rala*^AKO^ mice (Fig. 3f). We also noted that both basal and maximal respiration were higher in primary differentiated adipocytes from KO mice and the difference in maximal respiration was blunted by the addition of the CPT1 inhibitor etomoxir that blocks FAO (Fig. 4a and Extended Data Fig. 4a). To investigate directly whether RalA plays a role in controlling FAO, we incubated cells with (^14^C)-labeled palmitic acid (PA) and measured its oxidation to either acid-soluble metabolites (ASMs) or CO_2_ in WT and KO white adipocytes. In agreement with the OCR results, FAO was higher in KO compared to WT adipocytes (Fig. 4b). These data indicate that RalA KO in WAT increases energy expenditure due to increased mitochondrial oxidation activity.

To ensure that these studies reflected the activity of RalA, we also generated an immortalized preadipocyte line from *Rala*^f/f^ mice and induced *Rala* deletion by transducing cells with Cre lentivirus. The Cre recombinase completely ablated RalA in preadipocytes and fully differentiated adipocytes (Extended Data Fig. 4b). BODIPY staining demonstrated that both primary and immortalized preadipocytes from WT and KO mice were fully differentiated. As an orthogonal approach, we performed live-cell imaging using the cell permeant fluorescent dye, TMRM, to detect mitochondrial membrane potential (MtMP), which reflects electron transport and OXPHOS in active mitochondria. KO adipocytes exhibited a higher TMRM signal intensity than their WT counterparts (Fig. 4c and Extended Data Fig. 4c). To specify the ability of TMRM to detect mitochondrial depolarization in active mitochondria, we applied the β3-adrenergic receptor agonist CL-316,243 (CL) to induce mitochondrial membrane depolarization^32^. The TMRM signal declined quickly after administration of the agonist, which confirms that TMRM stains only active mitochondria (Extended Data Fig. 4d).

We previously reported that lipolysis drives mitochondrial oxidative metabolism in adipocytes^33^. To rule out a possible role for lipolysis as the primary driver of increased oxidative capacity of *Rala* KO adipocytes, we performed in vitro and in vivo lipolysis assays. CL robustly stimulated FFA and glycerol release to the same extent in KO and WT immortalized adipocytes and the molar ratio of FFA to glycerol was approximately 3:1 (Extended Data Fig. 4e,f). Additionally, there were no differences in CL-induced FFA and free glycerol production in *Rala*^f/f^ and *Rala*^AKO^ mice (Extended Data Fig. 4g,h). We also tested whether *Rala*^AKO^ mice are defective in the suppression of FFA release by insulin. Insulin suppressed CL-induced FFA release by approximately 50% in both WT and KO cells (Extended Data Fig. 4e). A single injection of insulin reduced FFA levels in control and *Rala*^AKO^ mice to the same extent (Extended Data Fig. 4i). Notably, KO adipocytes displayed a mild increase in glycerol release in the presence of CL, whereas *Rala*^AKO^ mice showed a mild decrease of plasma glycerol levels either in the presence of CL or after fasting (Extended Data Fig. 4f,h,j). Taken together, these results suggest that the absence of RalA in adipocytes enhances mitochondrial oxidative activity without affecting FFA supply.

### Targeted *Rala* knockout protects against obesity-induced mitochondrial fission in iWAT

**Extended Data Fig. 5:**
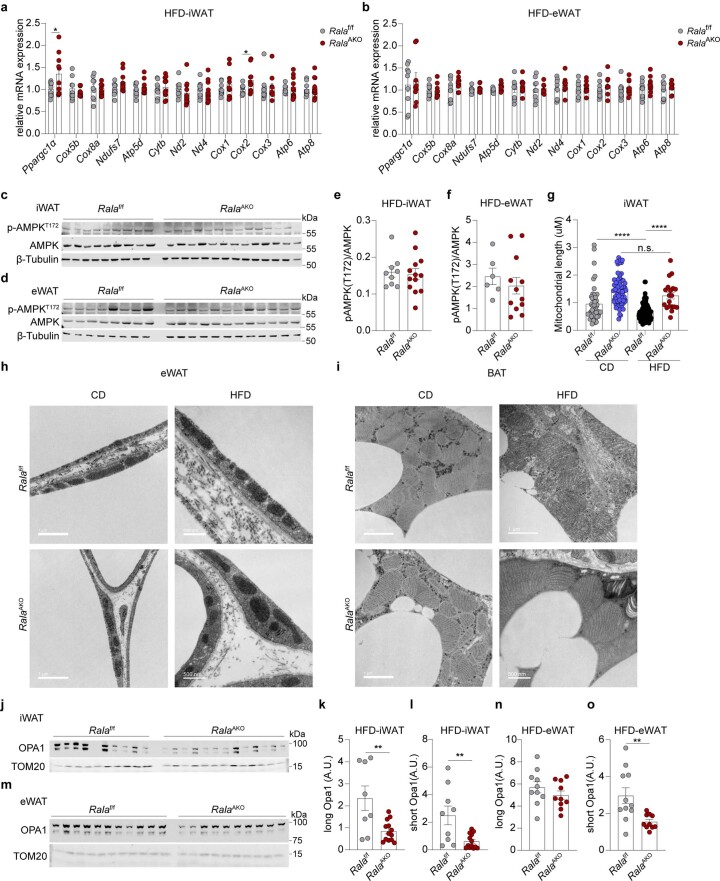
RalA inhibition did not affect mitochondrial biogenesis in WAT. **a**, **b**, Relative mRNA expression of genes corresponding to mitochondrial biogenesis in iWAT (**a**) and eWAT (**b**) of HFD-fed *Rala*^f/f^ (n = 9) and *Rala*^AKO^ (n = 10) mice. *P* = 0.0449, *P* = 0.0478. (**a**). **c**-**f**, Immunoblotting (**c**, **d**) and quantification (**e**, **f**) of phospho-AMPK (T172), total AMPK and β-Tubulin in iWAT (*Rala*^f/f^ n = 9, *Rala*^AKO^ n = 14) (**c**, **e**) and eWAT (*Rala*^f/f^ n = 8, *Rala*^AKO^ n = 12) (**d**, **f**) of HFD-fed mice. **g**, Maximal mitochondrial length in iWAT of CD-fed and HFD-fed mice (n = 3 biological samples). *P* < 0.0001, *P* < 0.0001, *P* = 0.1675. **h**, Representative TEM images of mitochondria in eWAT of HFD-fed mice, scale bar = 500 nm. **i**, Representative TEM images of mitochondria in BAT of HFD-fed mice, scale bar = 1 μm. **j**-**l**, Immunoblotting (**j**) and quantification (**k**, **l**) of Opa1 in iWAT of HFD-fed mice (*Rala*^f/f^ n = 10, *Rala*^AKO^ n = 13). *P* = 0.0045 (**k**). *P* = 0.0044 (**l**). **m**-**o**, Immunoblotting (**m**) and quantification (**n**, **o**) of Opa1 in eWAT of HFD-fed mice (*Rala*^f/f^ n = 14, *Rala*^AKO^ n = 10). *P* = 0.0063 (**o**). The data (**a**, **b**, **e**-**g**, **k**, **l**, **n**, **o**) are shown as the mean ± SEM, *P < 0.05, **P < 0.01, ****P < 0.0001 by two-tailed Student’s *T*-test (**a**, **g**, **k**, **l**, **o**). Source data

The increased mitochondrial oxidative activity observed in HFD-fed *Rala*^AKO^ mice could result from increased mitochondrial biogenesis. Expression of genes related to mitochondrial biogenesis was comparable between the genotypes (Extended Data Fig. 5a,b) in WAT. The activity of AMPK, the master regulator of mitochondrial biogenesis^34,35^, was also comparable between control and *Rala*^AKO^ mice fed with HFD (Extended Data Fig. 5c–f). In addition to biogenesis, mitochondrial function can also be regulated by dynamic changes in morphology through tightly controlled fusion and fission events that shape the organelle to comply with energy demands^19,36^. Electron microscopy (EM) revealed that HFD feeding of WT mice induced the appearance of smaller, spherical mitochondria in iWAT (Fig. 4d), consistent with previous reports that mitochondrial function and morphology is impaired in obese adipocytes^37,38^. We observed that mitochondria in iWAT changed from an elongated shape in CD-fed mice to a smaller size in HFD-fed mice (Extended Data Fig. 5g). Consistent with unaltered in vivo metabolic phenotypes, adipocyte *Rala* deletion did not grossly affect mitochondrial morphology in iWAT of CD-fed mice, but the HFD-induced change in mitochondrial morphology was completely prevented in *Rala* KO iWAT (Extended Data Fig. 5g). Indeed, tissue weight (Fig. 1g), OXPHOS content (Extended Data Fig. 3o,p) and mitochondrial OCR (Fig. 3f) were not affected by RalA deletion in eWAT, corresponding to the observation that the appearance of fragmented mitochondria in this depot was not reversed by *Rala* KO in HFD mice (Extended Data Fig. 5h). In fact, mitochondria in eWAT do not undergo significant fragmentation in response to HFD, possibly because of their already fragmented shape, consistent with the overall anabolic function of visceral adipocytes^39^. Moreover, mitochondrial morphology in BAT was not altered by RalA deletion in CD- or HFD-fed mice (Extended Data Fig. 5i). We also examined mitochondrial morphology in immortalized adipocytes differentiated from iWAT. As shown in Fig. 4e, mitochondria in KO adipocytes seemed longer than those in WT cells. There was a higher frequency of elongated mitochondria (1.0–1.5 μm) in KO cells (Fig. 4f) and the mean maximal mitochondrial length was significantly higher than in WT cells (Fig. 4g).

### Inhibition of RalA increases Drp1 S637 phosphorylation in white adipocytes

**Fig. 5:**
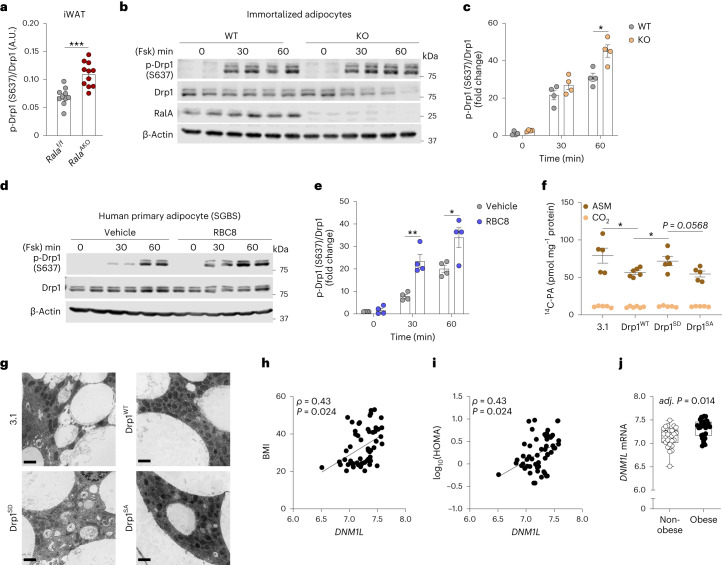
Inhibition of RalA increases Drp1 S637 phosphorylation in white adipocytes. **a**, Quantification of phospho-Drp1 (S637) and total Drp1 in iWAT of HFD-fed *Rala*^f/f^ (*n* = 10) and *Rala*^AKO^ (*n* = 13) mice; *P* = 0.0001. **b**,**c**, Immunoblotting (**b**) and quantification (**c**) of phospho-Drp1 (S637) and total Drp1 in immortalized adipocytes (*n* = 4 biological samples); *P* = 0.0125 (**c**). Adipocytes were treated with 20 μM forskolin (Fsk) for indicated time. **d**,**e**, Immunoblotting (**d**) and quantification of phospho-Drp1 (S637) and total Drp1 in human primary adipocytes (SGBS) (*n* = 4 biological samples). Cells were pretreated with 50 μM RBC8 or dimethylsulfoxide (DMSO) for 30 min before treatment with 20 μM forskolin (Fsk) for indicated time; *P* = 0.0022, *P* = 0.0244 (**e**). **f**, Basal ^14^C-PA oxidation in WT immortalized adipocytes transfected with indicated plasmids (*n* = 6 biological samples); *P* = 0.040 3.1 versus Drp1^WT^, *P* = 0.0364 Drp1^WT^ versus Drp1^SD^. **g**, Representative TEM images of immortalized adipocytes transfected with indicated plasmids (*n* = 3 biological samples). Scale bar, 2 μM. **h**,**i**, *DNM1L* mRNA expression is correlated with BMI (**h**) and HOMA (**i**) in human abdominal subcutaneous adipose tissue samples (*n* = 56 biological samples). *ρ* (rho) denotes Spearman’s rank-order correlation coefficient of the regression; *P* = 0.024 (**h**), *P* = 0.024 (**i**). **j**, Box-and-whisker plot of *DNM1L* mRNA expression in abdominal subcutaneous adipose tissues from 56 individuals with or without obesity. Benjamini–Hochberg adj. *P* = 0.014. The box plot is presented as a box showing 25th to 75th percentiles and whiskers showing min to max. Data (**a**,**c**,**e**,**f**) show mean ± s.e.m., *P < 0.05, **P < 0.01, ***P < 0.001 by two-tailed Student’s *t*-test (**a**,**c**,**e**,**f**). Significance in correlation was assessed by Spearman’s correlation test (**h**,**j**). Source data

**Extended Data Fig. 6:**
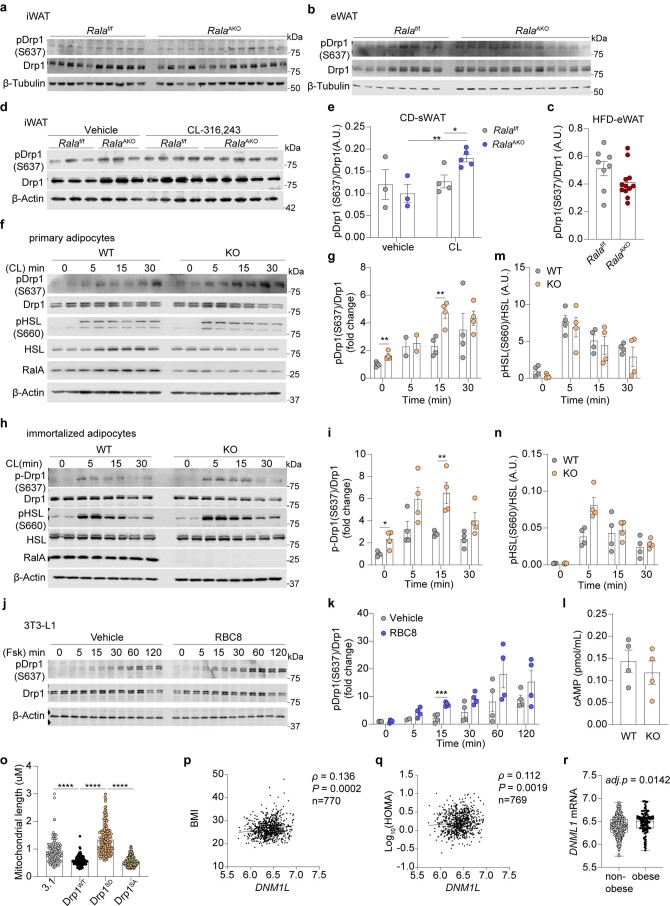
*Rala* deletion in adipocytes did not affect cAMP production and HSL phosphorylation. **a**, Immunoblotting of phospho-Drp1 S637, total Drp1 and β-Tubulin in iWAT of HFD-fed *Rala*^f/f^ (n = 10) and *Rala*^AKO^ (n = 13) mice. **b**, **c**, Immunoblotting (**b**) and quantification (**c**) of phospho-Drp1(S637), total Drp1, and β-Tubulin in eWAT of HFD-fed *Rala*^f/f^ (n = 8) and *Rala*^AKO^ (n = 12) mice. **d**, **e**, Immunoblotting (**d**) and quantification (**e**) of phospho-Drp1 (S637), total Drp1 and β-Actin in iWAT of CD-fed mice. Non-fasted *Rala*^f/f^ (vehicle n = 3, CL n = 4) and *Rala*^AKO^ (vehicle n = 3, CL n = 5) mice fed with CD were i.p. injected with 1 mg/kg CL for 30 min. *P* = 0.0054, *P* = 0.0135 (**e**). **f**, **g**, Immunoblotting (**f**) and quantification (**g**) of phospho-Drp1 (S637), total Drp1, RalA, and β-Actin in fully differentiated primary adipocytes (n = 4 biological samples). *P* = 0.0085, *P* = 0.0046 (**g**). Adipocytes were differentiated from stromal vascular fraction (SVF) isolated from 8-week-old female mice, and were treated with 1 μM CL for indicated time. **h**, **i**, Immunoblotting (**h**) and quantification (**i**) of phospho-Drp1 (S637), total Drp1, RalA, and β-Actin in immortalized adipocytes (n = 4 biological samples). *P* = 0.0159, *P* = 0.0086 (**i**). Adipocytes were treated with 1 μM CL for indicated time. **j**, **k**, Immunoblotting (**j**) and quantification (**k**) of phospho-Drp1 (S637), total Drp1, and β-Actin in 3T3-L1 adipocytes (n = 4 biological samples). *P* = 0.0009 (**k**). Cells were pretreated with 50 μM RBC8 or DMSO for 30 min, then treated with 5 μM forskolin (Fsk) for indicated time. **l**, Determination of intracellular cAMP levels in differentiated WT and *Rala*^AKO^ (KO) primary adipocytes (n = 4 biological samples). Cells were treated with CL for 5 min prior to harvesting. **m**, **n**, Quantification of phospho-HSL(S660) and HSL in primary (**m**) and immortalized adipocytes (**n**) treated with CL for indicated time (n = 4 biological samples). **o**, Maximal mitochondrial length in immortalized adipocytes expressing Drp1 mutants (n = 3 independent cells). *P* < 0.0001 3.1 vs Drp1^WT^, *P* < 0.0001 Drp1^WT^ vs Drp1^SD^, *P* < 0.0001 Drp1^SD^ vs Drp1^SA^. **p**-**r**, Transcriptomics and clinical data were directly accessed from GEO database (GSE70353). **p**, **q**, *DNM1L* mRNA expression is correlated with BMI (**p**) and HOMA (**q**) in human subcutaneous adipose tissue samples (n = 770). *P* = 0.0002 (**p**). *P* = 0.0019 (**q**). **r**, Box-and-whisker plot of *DNM1L* mRNA expression in abdominal subcutaneous adipose tissues from 770 individuals with or without obesity. Benjamini and Hochberg-adjusted *P* value (*adj. p*) is 0.014186. The box plot is presented as a box: 25^th^ to 75^th^ percentile, and whiskers: min to max. The data (**c**, **e**, **g**, **i**, **k**, **l**, **m**, **n**, **o**) are shown as the mean ± SEM, *P < 0.05, **P < 0.01, ***P < 0.001, ****P < 0.0001 by two-tailed Student’s *T*-test (**e**, **g**, **i**, **k**, **o**). Significance in correlation was assessed by Spearman’s correlation test (**p**, **q**). Source data

Opa1 and Drp1 have been identified as key regulators of mitochondrial fusion and fission, respectively^40^. Opa1 undergoes proteolytic cleavage to generate long (L-Opa1) and short (S-Opa1) forms that together fuel mitochondrial fusion^41–43^. Protein levels of both forms of Opa1 were downregulated in iWAT after HFD feeding (Extended Data Fig. 5j–l). Only S-Opa1 was downregulated in eWAT from *Rala*^AKO^ mice (Extended Data Fig. 5m–o), indicating the likelihood of reduced fusion in KO mice compared to WT littermates; however, the observation of elongated mitochondria in KO mice (Fig. 4d) suggests that this change in Opa1 processing is likely to be compensatory. We then focused on Drp1 as a key regulator of fission. Notably, Drp1 phosphorylation at the anti-fission S637 site was significantly increased in *Rala* KO iWAT (Fig. 5a and Extended Data Fig. 6a), whereas Drp1 S637 phosphorylation was comparable between the genotypes in eWAT (Extended Data Fig. 6b,c). Drp1 S637 phosphorylation is catalyzed by protein kinase A (PKA), activated by the β-adrenergic receptor–cAMP pathway^44,45^. To assess the role of RalA in modulating PKA action at this site, we assessed Drp1 S637 phosphorylation in iWAT. Phosphorylation was higher in CD-fed KO compared to WT mice in response to β-adrenergic stimulation (Extended Data Fig. 6d,e). This result ruled out the indirect regulation of Drp1 S637 phosphorylation by body weight differences in HFD-fed mice.

To establish whether this effect is cell-autonomous, we examined Drp1 phosphorylation in both immortalized and primary adipocytes. Consistent with in vivo results, *Rala* KO adipocytes showed a significantly higher Drp1 S637 after forskolin and β-adrenergic stimulation compared to WT cells (Fig. 5b,c and Extended Data Fig. 6f–i). We also explored the effect of RalA on Drp1 S637 phosphorylation state using a specific Ral inhibitor that prevents activation and retains GTPase in the GDP-bound, inactive state^26,46^. Pretreatment with the pan-Ral inhibitor RBC8 significantly increased forskolin-stimulated Drp1 S637 phosphorylation in 3T3-L1 adipocytes (Extended Data Fig. 6j,k). Inhibition of RalA activity with RBC8 also increased forskolin-stimulated Drp1 S637 phosphorylation in the human primary adipocyte cell line SGBS (Fig. 5d,e). To determine whether RalA influences CL-induced PKA activation or cAMP breakdown, we measured cAMP production and phosphorylation of hormone-sensitive lipase (HSL) in adipocytes. There were no differences in cAMP production between WT and KO primary adipocytes after 5 min of CL stimulation (Extended Data Fig. 6l). Similarly, HSL S660 phosphorylation was identical in WT and KO adipocytes (Extended Data Fig. 6m,n). Thus, RalA specifically modulates Drp1 S637 phosphorylation downstream of PKA activation across multiple adipocyte cell lines of both murine and human origin.

To investigate further whether Drp1 S637 phosphorylation is important for mitochondrial oxidative activity and morphology, we introduced S637 phospho-mimetic (SD) and phospho-null (SA) mutants into adipocytes and examined FAO and mitochondrial morphology. Cells expressing Drp1^SD^ had higher FAO than those expressing Drp1^WT^ and Drp1^SA^ (Fig. 5f). Consistent with this result, mitochondrial length in Drp1^SD^-expressing cells was higher than those with Drp1^WT^ and Drp1^SA^ expression (Fig. 5g and Extended Data Fig. 6o).

To examine the relevance of Drp1 as a regulator of metabolism in human obesity, we analyzed microarray data of abdominal subcutaneous WAT from obese and non-obese women. In human subcutaneous WAT, *DNM1L* (encoding human Drp1 protein) expression was positively correlated with body mass index (BMI) and HOMA-IR (Fig. 5h,i) and its expression was significantly upregulated in obese individuals (Fig. 5j), indicating that increased expression of *DNM1L* may contribute to mitochondrial dysfunction in obesity. Moreover, bioinformatic analysis of published microarray data (Gene Expression Omnibus (GEO) GSE70353) from 770 human males further confirmed that *DNM1L* is associated with obesity (Extended Data Fig. 6p–r). Together, these in vivo and in vitro data suggest that upregulated Drp1 activity in adipose tissue may be an important contributor to mitochondrial dysfunction during obesity, and further, that RalA deficiency protects mitochondria from excessive fission by increasing Drp1 S637 phosphorylation.

### RalA interacts with Drp1 and protein phosphatase 2A, promoting dephosphorylation of Drp1 at S637

**Fig. 6:**
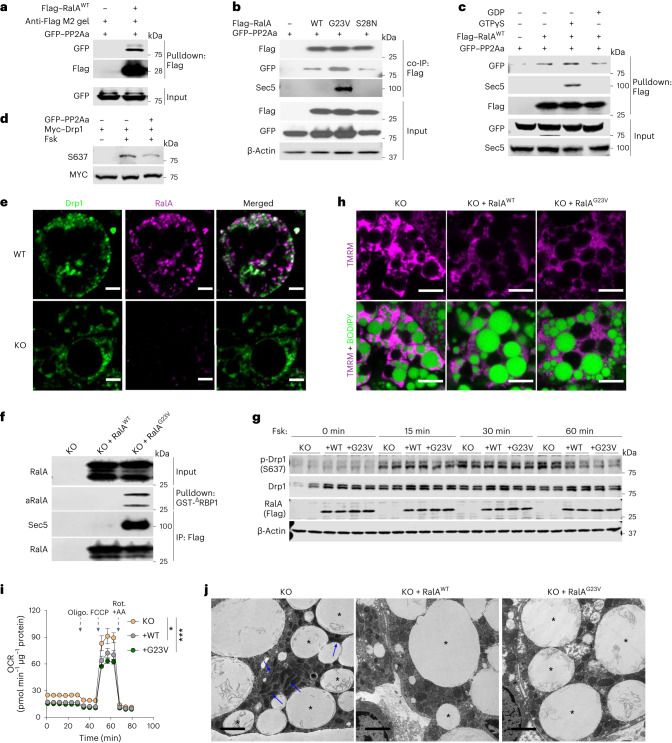
RalA interacts with Drp1 and protein phosphatase 2A, promoting dephosphorylation of Drp1 at S637. **a**, Representative immunoblotting of pulldown assay determining PP2Aa–RalA interactions. **b**, Representative immunoblotting of co-immunoprecipitation (co-IP) determining the interaction between RalA WT, constitutive active (G23V) or dominant negative (S28N) mutants and PP2Aa in HEK293T cells. **c**, Representative immunoblotting of pulldown and in vitro loading assay determining interaction between PP2Aa and GTP/GDP-loaded RalA. Purified Flag–RalA^WT^ protein loaded with either GTPγS or GDP was, respectively, used as a bait to pull down GFP–PP2Aa from HEK293T cells. **d**, Representative immunoblotting of in vitro dephosphorylation assay in HEK293T cells co-transfected with PP2A and Drp1 plasmids. Cells were treated for 1 h with 20 μM forskolin (Fsk) or vehicle. **e**, Representative immunofluorescent staining of endogenous Drp1 and RalA in immortalized WT adipocytes. Scale bar, 5 μm. **f**, Representative immunoblotting of RalA activity assay in immortalized *Rala* KO adipocytes reconstituted with RalA^WT^ and RalA^G23V^. **g**, Immunoblotting of phospho-Drp1 (S637), total Drp1, Flag-tagged RalA and β-actin in immortalized *Rala* KO adipocytes with or without RalA reconstitution (*n* = 3 independent experiments). Adipocytes were treated with 20 μM forskolin for the indicated times. **h**, Representative confocal images of live immortalized adipocytes (*n* = 3 biological independent cells) stained with TMRM (red) and BODIPY (green). Scale bar, 15 μM. **i**, OCR was measured by Seahorse in immortalized adipocytes (KO, *n* = 5 independent samples; +WT, *n* = 10 independent samples; +G23V, *n* = 9 independent samples); *P* = 0.0165 KO versus +WT, *P* = 0.0005 KO versus +G23V. Vertical arrows indicate injection ports of indicated chemicals. Data are shown as mean ± s.e.m., *P < 0.05, ***P < 0.001 by two-way ANOVA. **j**, Representative TEM images of *Rala* KO immortalized adipocytes with or without RalA reconstitution (*n* = 3 independent cells). Blue arrow indicates elongated mitochondria; asterisk indicates lipid droplet. Scale bar, 2 μm. Source data

**Extended Data Fig. 7:**
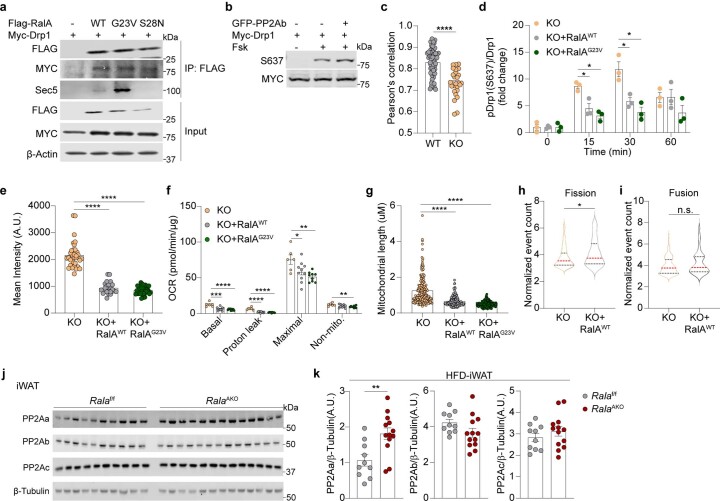
Knockout of RalA increases PP2Aa content. **a**, Representative immunoblotting of co-immunoprecipitated between Flag-RalA^WT^, Flag-RalA^G23V^, or Flag-RalA^S28N^ and Myc-Drp1 proteins in HEK293T cells. **b**, Representative *in vitro* dephosphorylation assay in PP2Ab and Drp1 co-transfected HEK293T cells treated with or without 20 μM forskolin (Fsk) for 1 hr. **c**, Quantification of Drp1 and RalA co-localization using Pearson’s method (n = 3 independent cells). *P* < 0.0001. **d**, Quantification of phospho-Drp1 (S637) and total Drp1 in immortalized RalA KO adipocytes with or without RalA reconstitution (n = 3 biological samples). 15 min: *P* = 0.0164 KO vs +WT, *P* = 0.0015 KO vs +GV. 30 min: *P* = 0.0185 KO vs +WT, *P* = 0.0087 KO vs +GV. Adipocytes were treated with 20 μM forskolin for indicated time. **e**, Quantification of TMRM fluorescence intensity in immortalized RalA KO adipocytes with or without RalA reconstitution (n = 3 independent cells). *P* < 0.0001 KO vs +WT. *P* < 0.0001 KO vs +GV. **f**, Calculated OCR in each state from immortalized RalA KO adipocytes with or without RalA reconstitution (WT = 6, +WT = 10, +GV = 9 biological samples). *P* < 0.0001, *P* = 0.0423, *P* = 0.0015, *P* = 0.0060. **g**, Maximal mitochondrial length in immortalized adipocytes (n = 3 independent cells). *P* < 0.0001 KO vs +WT. *P* < 0.0001 KO vs +GV. **h**, **i**, Quantification of fission (**h**) and fusion (**i**) events in immortalized adipocytes (KO = 92, +WT = 41). *P* = 0.0252 (**h**). *P* = 0.0879 (**i**). **j**, **k**, Immunoblotting (**j**) and quantification (**k**) of PP2Aa, PP2Ab, PP2Ac, and β-Tubulin in iWAT of HFD-fed mice (*Rala*^f/f^ n = 10, *Rala*^AKO^ n = 13). *P* = 0.0039 (**k**). The data (**c**, **d**, **e**, **f**, **g, k**) are shown as the mean ± SEM, *P < 0.05, **P < 0.01, ***P < 0.001, ****P < 0.0001 by two-tailed Student’s *T*-test (**c**, **d**, **e**, **f**, **g**, **h**, **k**). Source data

To understand the molecular mechanism by which RalA regulates Drp1 S637 phosphorylation, we used proteomics to search for proteins interacting with WT, constitutively active (G23V) or dominant negative (S28N) forms of RalA ectopically expressed in liver. Among the binding proteins was protein phosphatase 2A subunit Aα (PP2Aa), the scaffolding subunit encoded by the *Ppp2r1a* gene, which preferentially bound to the RalA^G23V^ constitutively active mutant. To confirm these mass spectrometry data, we purified RalA^WT^–Flag protein from HEK293T cells and pulled down PP2Aa from lysates (Fig. 6a). To determine whether this interaction is dependent on the activation state of the G protein, we coexpressed WT and mutant RalA constructs with PP2Aa in HEK293T cells. As a positive control, the effector Sec5 only bound to active RalA^G23V^ (ref. ^47^). Similarly, this mutant form of RalA had the highest affinity for PP2Aa (Fig. 6b). We also loaded a RalA–Flag fusion protein in vitro with GTPγS or GDP to evaluate the specificity of effector binding^22^. Both Sec5 and PP2Aa were pulled down by RalA loaded with GTPγS but not with GDP (Fig. 6c). In addition, because PP2Aa and Drp1 did not independently interact (data not shown), we investigated whether RalA directly modifies Drp1 phosphorylation via PP2Aa. When coexpressed, Drp1 and RalA interacted directly with each other, although there was no preference for the activation state of RalA (Extended Data Fig. 7a). Activation of the cAMP–PKA axis by addition of forskolin increased Drp1 S637 phosphorylation, whereas coexpression of PP2Aa promoted the dephosphorylation of S637 (Fig. 6d), although overexpression of PP2Ab had no effect (Extended Data Fig. 7b). These data suggest that Drp1 is constitutively associated with RalA independent of activation state and upon activation, RalA recruits PP2Aa to promote the dephosphorylation of Drp1 S637.

Drp1 colocalized with RalA In adipocytes and this colocalization was not observed in RalA KO adipocytes (Fig. 6e and Extended Data Fig. 7c). To understand further the effects of RalA activation state on Drp1 phosphorylation and mitochondrial function, we transduced immortalized RalA KO cells with RalA^WT^ and RalA^G23V^ lentivirus before differentiation into adipocytes. RalA^G23V^-expressing adipocytes showed a robust increase in RalA–GTP binding (Fig. 6f) and these cells had significantly less Drp1 S637 phosphorylation (Fig. 6g and Extended Data Fig. 7d). Expression of either RalA^WT^ or RalA^G23V^ significantly reduced mitochondrial potential in KO adipocytes (Fig. 6h and Extended Data Fig. 7e). To confirm that this reduction in mitochondrial potential is associated with reduced oxidative function, we performed a Seahorse assay. Consistent with results in primary adipocytes, RalA^WT^- and RalA^G23V^-expressing adipocytes displayed reduced basal and maximal OCR in comparison to KO adipocytes (Fig. 6i and Extended Data Fig. 7f). In addition, EM revealed that overexpression of WT or constitutively active RalA in adipocytes resulted in fragmented mitochondria, indicating increased fission compared to RalA KO adipocytes (Fig. 6j and Extended Data Fig. 7g). Live-cell imaging analyses also indicated fewer fission events in KO compared to WT adipocytes, whereas no differences were detected in fusion (Extended Data Fig. 7h,i and Supplementary Videos 1 and 2).

**Extended Data Fig. 8:**
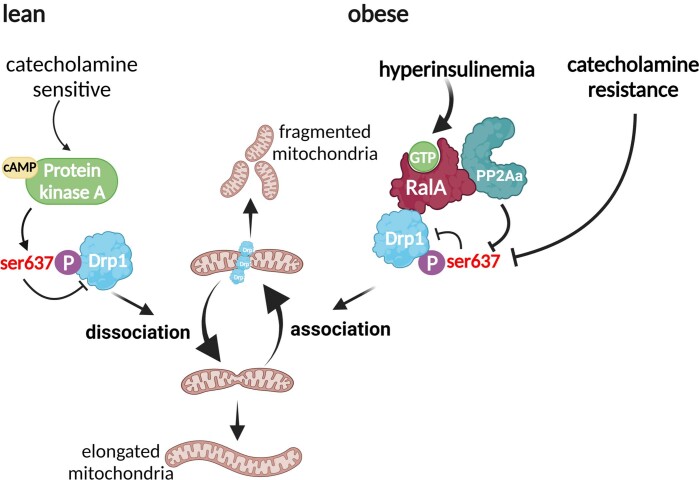
Mechanistic model depicting how RalA regulates mitochondrial function in obese adipocytes. Created with BioRender.com. Obesity drives RalA expression and GTP binding activity, leading to its association with PP2Aa, which in turn recruits the catalytic subunit PP2Ac to dephosphorylate Drp1 S637. Also, catecholamine resistance could reduce PKA-catalyzed S637 phosphorylation. The combined effects converging on RalA-PP2A-Drp1signaling axis result in constitutive mitochondrial translocation of Drp1 and fragmented mitochondria in adipocytes from obese subjects.

RalA has previously been reported to promote fission in proliferating cells and *Rala* knockdown led to a long, interconnected mitochondrial network and reduced proliferation^48^. Partially in agreement with this study, we found that RalA deficiency resulted in elongated mitochondria in adipocytes, with increased OXPHOS that dramatically impacted whole-body lipid metabolism; however, unlike the previous study, we did not observe an interaction between RalBP1 and Drp1. Notably, total PP2Aa protein levels were increased in *Rala* KO compared to control iWAT, without a difference in PP2Ab and PP2Ac content (Extended Data Fig. 7j,k), perhaps reflecting a compensatory pathway. Taken together, our data suggest that obesity drives RalA expression and GTP binding activity, leading to its association with PP2Aa, which in turn recruits the catalytic subunit PP2Ac to dephosphorylate Drp1 S637. We also note that catecholamine resistance, an inherent trait of the obese state^28^, is also expected to lead to reduced PKA-catalyzed S637 phosphorylation. Together, these effects result in constitutive mitochondrial translocation of Drp1 and fragmented mitochondria in adipocytes from obese individuals (Extended Data Fig. 8).

## Discussion

While accumulating evidence suggests that mitochondrial dysfunction is a characteristic trait of obesity in human and rodent adipocytes^16,37,38,49^, the underlying molecular mechanisms remain unknown. Here, we report a new regulatory axis for the control of mitochondrial morphology and function in the context of obesity, involving prolonged activation of the small GTPase RalA. We show that RalA is both induced and activated in white adipocytes after feeding rodents HFD, whereas the negative regulator of RalA, RalGAP, is downregulated. We also observe a positive correlation of expression of the RalGEF RGL2 with BMI in adipose tissue of humans with obesity, expected to correspond to a chronic increase in RalA activity. The increase in adipocyte RalA messenger RNA, protein and activity is associated with mitochondrial dysfunction, characterized by fragmentation and reduced oxidative capacity, specifically in iWAT. Targeted deletion of RalA in white adipocytes prevents the obesity-dependent fragmentation of mitochondria and produces mice resistant to HFD-induced weight gain via increased energy expenditure. In vitro studies revealed that RalA suppresses mitochondrial oxidative function in adipocytes by increasing fission through reversing the inhibitory phosphorylation of the mitochondrial fission protein Drp1. This reduced phosphorylation results from the recruitment of the regulatory subunit of PP2A, which acts as a bona fide effector of RalA, leading to the specific dephosphorylation of the inhibitory Ser^637^ residue on Drp1, rendering the protein active. We also note our previous study in which constitutive activation of RalA via adipocyte-specific KO of *Ralgapb* produced a significant enlargement of white adipocytes and increased adipose tissue mass, even on a control diet^26^. Thus, chronic elevation in RalA activity plays a key role in repressing energy expenditure in obese adipose tissue, contributing to weight gain and related metabolic dysfunction, including glucose intolerance and fatty liver, and may explain in part how energy expenditure is repressed in prolonged obesity^50^.

The observation that adipocyte RalA controls overall systemic metabolism via this mechanism was noteworthy. We, and others, previously reported that RalA plays a key role in controlling the trafficking of GLUT4 vesicles in adipocytes and muscle^22,23^. RalA is activated by insulin, mainly by inhibition of its GAP complex through phosphorylation^24,51^ and when activated, RalA interacts with components of the exocyst complex to target GLUT4 vesicles to the plasma membrane for fusion, increasing glucose uptake into fat cells^21^. Indeed, adipocytes treated with an RalA inhibitor^26^ or isolated from RalA KO mice showed dramatically reduced GLUT4 translocation to the plasma membrane, with less glucose uptake in response to insulin. Targeted deletion of the scaffolding subunit of the RalGAP complex resulted in constitutive activation of RalA in adipocytes and myocytes and dramatically improved glucose homeostasis^22,26,51^; however, detailed physiological tracer studies revealed that improvements in glucose disposal in adipocyte-specific KO mice occurred primarily in brown fat, where glucose uptake was markedly increased^26^. Consistent with these findings, we observed that *Rala* deletion reduces glucose uptake mainly in BAT, with a smaller effect in iWAT and no effect in eWAT. We also saw a small reduction in insulin sensitivity in *Rala*^AKO^ mice on a control diet, accompanied by reduced weights of all adipose tissues, likely reflecting less nutrient uptake; however, *Rala*^AKO^ mice on HFD paradoxically showed improved glucose tolerance and insulin sensitivity. While it remains unclear exactly how these mice overcome the negative effects of RalA deletion on glucose uptake, GLUT4 mRNA and protein levels in WAT are downregulated in obesity^12,52,53^, whereas GLUT1 mRNA and protein levels are increased^54,55^, consistent with our RalGAP KO studies in HFD-fed mice that show little glucose uptake into white fat in response to insulin, but higher basal levels^26^. Additionally, brown fat develops insulin resistance in HFD mice, accompanied by an overall reduction in RalA activity even before KO. Thus, it seems likely that improved glucose tolerance in *Rala*^AKO^ mice occurs because of increased energy expenditure primarily from FAO.

It was also notable that liver function was dramatically improved in *Rala*^AKO^ mice on HFD, with reduced hepatic lipids and gluconeogenesis, as indicated by improvements in pyruvate tolerance. It is well established that WAT plays an important role in regulating whole-body energy metabolism^56^. Hepatic acetyl-CoA arises from WAT lipolysis to directly promote hepatic gluconeogenesis^57^. The increase in FAO in *Rala* KO adipocytes resulted in fewer circulating FFAs and TGs, likely producing improved liver health and reduced gluconeogenesis.

While the significance of the adipose depot specificity of the effects of RalA remains uncertain, we note that adipocytes in visceral, subcutaneous and brown fat differ in many ways^58,59^. Although RalA was deleted in all adipocytes in *Rala*^AKO^ mice, mitochondrial function was only improved in iWAT. While there are numerous differences between visceral and inguinal white adipocytes that might explain this, including their response to HFD, one notable issue has to do with inherent mitochondrial morphology. Upon HFD feeding, adipocytes in iWAT underwent a dramatic size expansion, accompanied by a change in mitochondria from an elongated to a fragmented morphology, reflecting a transition to a largely anabolic state. These changes were not observed in RalA KO mice. Unlike what was observed in iWAT, mitochondria in eWAT display a fragmented morphology even in lean mice, with no change observed after HFD or RalA KO, consistent with the overall energy storage function of this depot even without the anabolic pressure of overnutrition.

Another question concerns the role of RalA in BAT. While BAT tissue weight was reduced in both *Rala*^AKO^ and *Rala*^BKO^ mice compared to controls, likely due to reduced glucose uptake, only iWAT adipocytes seem to respond with a change in metabolic activity and mitochondrial morphology. Brown adipocyte mitochondria are morphologically different from those in white adipocytes; brown adipocyte mitochondria are more numerous and larger than the mitochondria in white adipocytes and contain packed cristae. Comparison of the mitochondria of brown and white adipocytes by proteomic analysis revealed that proteins involved in pathways related to fatty acid metabolism, OXPHOS and the tricarboxylic acid cycle were highly expressed in BAT compared to WAT^60^. Thus, it seems likely that mitochondria in BAT are subjected to fundamentally different modes of regulation than those in white fat, and the reduced weight of BAT in KO mice can be attributed to reduced glucose uptake.

As mitochondrial function is vital for healthy metabolism, efforts have focused on preventing fragmentation via blocking the activity or direct deletion of Drp1 (ref. ^61^). Muscle mitochondrial dysfunction is closely related to excessive Drp1 activity^62^ and elevated Drp1 activating S616 phosphorylation has been found in severely obese human muscle^63,64^. On the other hand, triggering Drp1 S637 phosphorylation has been suggested to increase the uncoupling capacity of FFA in brown adipocytes^65^. In line with this observation, increased S637 phosphorylation was found in BAT after cold exposure^66^. Administration of a Drp1 inhibitor acutely improved muscle insulin sensitivity and systemic glucose tolerance^67,68^; however, the impact of modulating Drp1 levels and phosphorylation states is complicated and varies between tissues. Targeted deletion of Drp1 in liver reduced hepatic lipid accumulation and body weight in a non-alcoholic fatty liver disease model^69^. Moreover, loss of Drp1 impairs brown adipocyte differentiation and thermogenesis, possibly reflecting the aspects of mitochondrial morphology that are unique to BAT^66,70^. In this regard, an association of S637 phosphorylation with fission was reported in brown adipocytes, although it remains possible that increased fission might reflect the phosphorylation of the activating S616 site^65,71^. Moreover, mitochondrial fission not only leads to increased oxidative metabolism but also triggers mitophagy to clear damaged mitochondria^72^. Notably, endoplasmic reticulum stress has been observed in tissue-specific Drp1 KO mice models, which suggests that Drp1 may also regulate endoplasmic reticulum remodeling^73^. These findings highlight the likely differences between total ablation of Drp1 activity and changes in its upstream regulatory pathways.

Many questions remain concerning the role of the RalA–Drp1 axis in the control of mitochondrial function in subcutaneous adipocytes. What is the mechanism by which RalA mRNA and protein expression are increased and RalGAP is decreased in adipose tissue during obesity? Additionally, the factors leading to increased RalA–GTP binding are not known, although this may be a result of reduced RalGAP expression, as well as hyperinsulinemia and chronic elevations in Akt activity seen in obesity^74^. A key question concerns the spatial compartmentalization of RalA activation and Drp1 phosphorylation/dephosphorylation in adipocytes. Are there pools of RalA in different cellular compartments that interact with different effectors? Do other isoforms of RalA (RalB) also control Drp1 localization and function? What is the domain of PP2Aa that interacts with RalA? While many questions remain, these findings open a new line of investigation concerning how the RalA–Drp1 axis regulates energy homeostasis.

## Methods

### Ethical statement

The animal study was approved by the Institutional Animal Care and Use Committee (IACUC) at the University of California, San Diego (UCSD). We obtained human data from a third-party database and collaborator. Human studies were approved by the corresponding institutes and informed written consent was provided by the participants. The cell culture study was approved by the Environment, Health and Safety Department at UCSD.

### Animals

RalA-floxed (*Rala*^f/f^) mice were bred with adiponectin-promoter-driven Cre or Ucp1-promoter-driven Cre transgenic mice to generate fat depot-specific RalA KO (*Rala*^AKO^ or *Rala*^BKO^) mice. All mice were on a C57BL/6J background and all experiments were performed using littermates. Male mice were used for in vivo experiments and female mice were used only for primary preadipocyte isolation. We fed mice with standard CD (Teklad, 7912) or HFD consisting of 60% calories from fat (Research Diets, D12492) for 8–12 weeks, starting from 8 weeks of age. Mice were housed in a specific-pathogen-free facility with a 12-h light–dark cycle and given free access to food and water, except for the fasting period. The facility temperature and humidity were constantly kept at 22 °C and 50%. All animal experiments were approved by and followed the guidelines from the IACUC at the UCSD.

### Cell culture

#### Primary preadipocytes

Inguinal WAT from 2–3 8-week-old female mice was dissected, minced and digested in 5 ml 1 mg ml^−1^ collagenase (Sigma) for 15 min (min) in a 37 °C water bath with gentle agitation. DMEM/F12 medium (15 mM HEPES) with 10% FBS (growth medium) was added to stop digestion and cells were filtered through 100-μm and 70-μm strainers. After centrifugation at 750*g*, cells were plated onto dish with growth medium. Once cells reached 90% confluence, preadipocytes were seeded into 12-well plates or imaging dishes for differentiation. Differentiation was induced in growth medium containing 0.5 mM IBMX, 5 μM dexamethasone, 1 μM rosiglitazone and 5 μg ml^−1^ insulin for 3 d. Medium was then switched to growth medium with rosiglitazone (day 3–5) and insulin (day 3–7). From day 7, cells were maintained in growth medium until they were 100% differentiated.

#### Immortalized adipocytes

Primary preadipocytes from *Rala*^f/f^ mice were immortalized by retroviral transduction of pBabe-zeo-LT-ST(SV40) and selection by Zeocin^75^. Single-cell clones were selected and tested for differentiation capacity. All used clones in this study displayed 100% adipocyte morphology after differentiation. To generate *Rala* KO cells, immortalized *Rala*^f/f^ (WT) preadipocytes were transduced with lentiviral Cre with 8 μg ml^−1^ Polybrene for 12 h, then cultured in DMEM/F12-FBS medium. Cre recombinase efficiency was tested in preadipocytes and adipocytes. Once reaching 95–100% confluence (day 0), differentiation was induced as described above. On the day of experiment, cells were starved in DMEM/F12 medium 3 h before treatments.

#### Human primary preadipocytes (SGBS)

Cells were cultured in DMEM/F12-FBS medium supplemented with 3.3 mM biotin (Sigma, B4639) and 1.7 mM pantothenate (Sigma, P5155) and differentiated with a published protocol^76^.

#### 3T3-L1 adipocytes

Preadipocytes were cultured in high-glucose DMEM with 10% newborn calf serum (culture medium). At 2 d after confluency, differentiation was induced in culture medium containing 0.5 mM IBMX, 5 μM dexamethasone and 2 μg ml^−1^ insulin for 3 d. The medium was then switched to growth medium with insulin (day 3–7) or without insulin (day 7 to fully differentiated).

#### Lenti-X 293T cells

Lenti-X 293T cells were cultured in high-glucose DMEM-FBS medium for packing lentivirus. When cells reached 100% confluency on a 0.01% poly-lysine-coated dish, third-generation lentiviral packaging plasmids (pLVX vectors, pMDLg/pRRE (Addgene #12251), pRSV-Rev (Addgene #12253) and pMD2.G (Addgene #12259)) were transfected into cells using lipofectamine 3000 (Life Technology) following the manufacturer’s protocol. Fresh DMEM-FBS medium with 25 mM HEPES was added 12–16 h after transfection. The lentivirus-containing medium was collected twice at 48 and 72 h after transfection. After collection, the medium was spun at 300*g* for 5 min to remove dead cells, then incubated with Lenti-X concentrator (Takara) at a 3:1 ratio at 4 °C overnight. The viral pellets were collected by centrifugation at 1,500*g* for 45 min at 4 °C and reconstituted in DMEM/F12-FBS medium with 8 μg ml^−1^ Polybrene. Lentivirus was added to cells immediately after reconstitution.

#### Reconstitution of RalA^WT^ and RalA^G23V^ in RalA KO preadipocytes

Immortalized RalA KO preadipocytes were transduced with concentrated Flag–RalA^WT^ or Flag–RalA^G23V^ lentiviral supernatants with 8 μg ml^−1^ Polybrene. At 24 h after infection, the medium was changed to fresh DMEM/F12-FBS and expanded for differentiation. Expression of Flag-tagged protein was examined in fully differentiated cells by western blot.

### Gene analysis in clinical cohorts

The transcriptomics data from abdominal subcutaneous WAT of 30 individuals with obesity and 26 healthy women were generated as previously described^77^. Transcriptome profiles were obtained using GeneChip Human Gene 1.0 ST Arrays. Data were deposited in the NCBI GEO under accession code GSE25402. Transcriptome profiles in the verification cohort were obtained from subcutaneous fat biopsies from 770 men participating in the METSIM study^78^. Transcriptomics and clinical data were retrieved from GEO (GSE70353). Obesity is defined as a BMI > 30 kg m^−2^ in these analyses.

### Primary mature adipocyte isolation

Minced WAT was digested in DMEM with 1 mg ml^−1^ collagenase (Sigma) for 25 min at 37 °C with gentle agitation. The cell suspension was filtered through a 100-μm cell strainer and centrifuged at 50*g* for 3 min to separate floating mature adipocytes. Floating mature adipocytes were transferred to PBS with broad open tips and washed twice. Then, 1 ml mature adipocytes were lysed in 4 ml TRIzol (Life Technology) for RNA isolation.

### RNA sequencing analysis

RNA extractions from primary mature inguinal and epididymal adipocytes were performed using TRIzol (Life technologies) and PureLink RNA mini kit (Life Technologies), according to the manufacturer’s instructions. RNA quality was checked by an Agilent TapeStation. Biological triplicates of isolated 500 ng RNA were used to prepare sequencing libraries using the TruSeq RNA Sample Preparation kit v.2 (Illumina), according to the manufacturer’s protocol. Libraries were validated using a 2100 BioAnalyzer (Agilent), then normalized and pooled for sequencing using bar-coded multiplexing at a 90-bp single-end read length on an Illumina HiSeq 4000. Samples were sequenced to a median depth of 14 million reads.

### Bioinformatics analysis

For RNA-seq, sequencing fastq files were generated automatically using the Illumina bcl2fastq2 Conversion Software. Read alignment and junction mapping to genome mm39 (GRCm39) and the mouse Genecode M30 annotation were accomplished using STAR (v.2.7.2b). Known splice junctions from mm10 were supplied to the aligner and de novo junction discovery was also permitted. Differential gene expression analysis and statistical testing were performed using DESeq2 with an adjusted *P* value < 0.05 as a cutoff. Raw gene counts were normalized to fragments per million mapped fragments (FPM) using DEseq2. FPM counts were filtered, centered by *z* score before gene clustering and heat map generation using GENE-E (v.3.0.215) or GraphPad Prism (v.8.4.3). For microarray data, gene matrix files were collapsed using the Collapse Dataset tool in GSEA (v.4.3.2) using chip platform (GPL13667) with collapsing mode (Mean_of_probes). The statistical significance of differential gene expression was assessed by ComparativeMarkerSelection module (v.11) from GenePattern (https://cloud.genepattern.org/gp/pages/index.jsf).

### Gene expression analysis

Tissue RNA was isolated with TRIzol reagent in combination with column (PureLink RNA mini, Invitrogen) according to the manufacturer’s protocol. Complementary DNA was generated from 1 μg RNA using the cDNA Maxima Reverse Transcription kit (Thermo Fisher Scientific). mRNA expression was assessed by real-time PCR using the QuantStudio real-time PCR system and SYBR Green PCR master mix (Invitrogen). Gene expression was normalized to *Cyclophilin A* in murine tissues. Relative mRNA expression levels were calculated using averaged 2^−ΔΔCt^ values for each biological replicate. Primers are listed in Extended Data Table 1.

### Protein isolation and western blotting

Tissue or cells were lysed or homogenized in RIPA buffer with freshly added Halt Protease and Phosphatase Inhibitor Cocktail (Thermo Fisher). Lysates were rotated in a cold room for 30 min, then briefly sonicated and centrifuged at 17,000*g* for 15 min at 4 °C. Cleared supernatants were collected and concentrations were determined with a BCA protein assay kit (Pierce) and iTecan plate reader for quantification. Proteins were resolved by Tris-Glycine gel (Novex, Invitrogen) electrophoresis and transferred to nitrocellulose membranes. Individual proteins were detected with the specific antibodies (OXPHOS ab110413, β-tubulin 2146S, phospho-Drp1(Ser637) 4867S, phospho-HSL(Ser660) 45804S, HSL 4107S, MYC 2276S, Drp1 8570S, phosphor-AMPK(Thr172) 2535S, AMPK 5831S, RalA BD610221, β-actin 66009-1-Ig, Flag 66008-4-Ig, GFP 66002-1-Ig and Sec5 12751-1-AP) and visualized on blots using fluorescent secondary antibodies with a Li-Cor system or on film using HRP-conjugated secondary antibodies (Fisher Scientific) with SuperSignal West Pico Chemiluminescent substrate (Thermo Fisher). All primary antibodies were used at 1:1,000 dilution, fluorescent secondary antibodies were used at 1:5,000 dilution and HRP-conjugated secondary antibodies were used at 1:8,000 dilution. Bands were quantified with ImageStudio or ImageJ.

### Body-mass composition

Body-mass composition was assessed in non-anesthetized mice using EchoMRI.

### Glucose tolerance test

Mice were fasted for 6 h, then intraperitoneally (i.p.) injected with d-[+]-glucose in PBS at a dose of 2 g kg^−1^ BW for CD-fed mice or 1.2 g kg^−1^ BW for HFD-fed mice. Blood glucose levels were measured before injection and at 15, 30, 60, 90 and 120 min after injection using the Easy Touch glucose monitoring system.

### Insulin tolerance test

Mice were fasted for 4 h, then i.p. injected with human insulin (Sigma) in saline at a dose of 0.35 U kg^−1^ BW for CD-fed mice or 0.6 U kg^−1^ BW for HFD-fed mice. Blood glucose levels were measured as described above.

### Pyruvate tolerance test

Mice were fasted for 16 h, then i.p. injected with pyruvate in PBS at a dose of 1.5 g kg^−1^ BW for HFD-fed mice. Blood glucose levels were measured as described above.

### Blood parameters

Whole blood was taken from the facial vein and blood glucose was measured with a glucose meter (Easy Touch) from the tail vein. Plasma was collected after centrifugation at 1,200*g* at 4 °C for 10 min. Plasma TG and FFA levels were measured with an Infinity Triglycerides kit (Thermo Fisher) and NEFA kit (WAKO). Plasma insulin levels were measured with the Mouse Ultrasensitive Insulin ELISA kit (Crystal Chem, 90080) and leptin levels were measured with a Mouse Leptin ELISA (Crystal Chem, 90030) kit. Plasma AST and ALT activity was measured with the Aspartate Aminotransferase Activity kit (Biovision, K753) and Alanine Aminotransferase Activity kit (Biovision, K752).

### HOMA-IR calculation

HOMA-IR is an index of overall insulin sensitivity^79^. Glucose and insulin levels from overnight-fasted mice were measured as described above. The values were used to calculate HOMA-IR with the formula: fasting insulin (μU l^−1^) × fasting glucose (nmol l^−1^)/22.5.

### Hepatic lipid TG measurement

Frozen liver tissue (50–100 mg) was homogenized in 1 ml PBS. Then, 800 μl lysates were added to 4 ml extraction buffer. After thoroughly rotating for 30 min at room temperature (RT), the lipid phase was separated from the aqueous phase by centrifuging at 1,800*g* for 20 min. A 0.2-ml lipid fraction in the organic phase was collected and transferred to a 1.5-ml tube to dry under a nitrogen stream in the fume hood. Then, 0.2 ml 2% Triton X-100 solution was used to solubilize the lipids. TG levels were determined using the Infinity Triglycerides kit (Thermo Fisher). The lipid amount was normalized to the liver lysate protein amount.

### Histology

For H&E staining, liver tissue was collected and fixed in 10% formalin. Paraffin-embedding, sectioning and H&E staining was completed at the UCSD Biorepository and Tissue Technology Shared Resources (BTTSR). For adipocyte size quantification, H&E slides were imaged using a Keyance brightfield microscope or a Nikon confocal microscope with Texas Red excitation and emission filters. Adipocyte size was assayed using Adiposoft in ImageJ and an in-house-developed pipeline with Cell Profiler. For Oil-Red-O staining, liver tissue was fixed in 4% PFA at 4 °C for 24 h, then transferred to 20% sucrose/PBS for 24 h. Afterwards, tissue was embedded in O.C.T. (Sakura) with dry ice and ethanol. Frozen tissue blocks were sectioned and stained with Oil-Red-O at the UCSD BTTSR.

### Indirect calorimetric measurements

For metabolic cage studies, mice were individually housed in Promethion metabolic cages maintained at 22 °C under a 12-h light–dark cycle. Before the experiment, mice were adapted to the metabolic cages for 2 d. The monitoring system records and calculates food intake, locomotor activity, oxygen consumption, CO_2_ production, RER and EE. Mice were provided with free access to water and food during the entire measurement. The data were exported with ExpeData software (Sable Systems) and EE was analyzed using ANCOVA with BW as a covariate by a web-based CalR tool^80^.

### Respiration measurement

#### Intact cells

The cellular OCR was measured using an eXF96 Extracellular Flux Analyzer and analyzed by Agilent Seahorse Wave Software (Seahorse Bioscience). Before assay, 2,500 primary preadipocytes were seeded and differentiated in XF96 microplates. Once fully differentiated, adipocyte culture medium was changed to assay medium containing 25 mM glucose, 1 mM pyruvate and 2 mM l-glutamine and 0.5 mM carnitine without phenol red or sodium bicarbonate for 3 h. Before the measurement, cells were incubated in a CO_2_-free incubator for 15 min. Basal rates of respiration were measured in assay medium and followed with sequential injections of oligomycin (2 μM), FCCP (0.5 μM) and rotenone with antimycin A (each 0.5 μM). Oxygen consumption values were normalized to protein content.

#### Isolated mitochondrial

Isolation of mitochondrial from HFD-fed mice and the OCR with 2.5 μg isolated mitochondrial was performed according to our published protocol^33^.

### Fatty acid oxidation assay

Fully differentiated primary adipocytes in 24-well plates were incubated in 0.5 ml DMEM per well containing 1 mM carnitine and 0.5 μCi per well and [^14^C]-PA for 60 min at 37 °C. Afterwards, 360 μl medium was collected and added to 40 μl 10% BSA in a 1.5-ml tube with a filter paper in the cap. Then, 200 μl 1 M perchloric acid was added to the tube and the cap was immediately closed tightly and incubated at RT. After 1 h, captured CO_2_ and ASMs were used to measure radioactivity. The cells were lysed in NaOH/SDS buffer (0.3 N/0.1%) to measure protein concentration. FAO rates were normalized to protein content.

### Glucose uptake assay

#### In vivo

CD-fed mice were fasted for 6 h and 10 μCi [^3^H]-deoxy-glucose or [^14^C]-deoxy-glucose was i.p. injected alone or spiked with 1.2 g kg^−1^ glucose into each mouse. Then, 30 min after injection, plasma and tissues were collected and snap frozen until further processing. The accumulation of deoxy-glucose-phosphate in different tissues was determined using a published protocol^26^.

#### In vitro

Fully differentiated primary adipocytes were fasted in serum-free medium for 3 h before the assay. A glucose uptake-Glo assay was performed according to the manufacturer’s protocol (Promega).

### Confocal microscope imaging

#### Live cells

Fully differentiated adipocytes were cultured in a glass-bottom dish (Cellvis) and incubated in phenol-red-free DMEM (imaging medium) with 100 nM TMRM (Thermo Fisher) for 30 min to indicate mitochondrial membrane potential and BODIPY 493/503 (final 5 μg ml^−1^, Life Technology) was added to label lipid droplets for the last 15 min. Cells were then washed three times with imaging medium. Live-cell images were obtained with a Nikon A1R confocal microscope with ×100 or ×60 oil immersion objectives. For time-lapse imaging, pictures were taken every 10 min.

#### Fixed cells

Fully differentiated primary adipocytes were cultured in a glass-bottom chamber (Lab-Tek). On the day of the experiment, cells were serum-starved for 3 h and treated with 100 nM insulin. After 15 min, the medium was removed, cells were fixed with ice-cold methanol and incubated at −20 °C for 10 min. Cells were then washed twice with PBS and blocked with 10% goat serum in PBS with 0.1% Triton X-100 at RT for 30 min. After blocking, cells were incubated with primary antibodies (1:50 dilution) at 4 °C overnight and secondary antibodies (1:2,000 dilution) for 1 h at RT. Cells were washed three times with PBS before imaging with a Nikon A1R confocal microscope using a ×100 oil immersion objective.

### 4D mitochondria live-cell imaging and analysis

A custom-built lattice light-sheet microscope designed by the Betzig Laboratory HHMI Janelia/UC Berkeley was used to image fully differentiated adipocytes^81^. The 488-nm and 560-nm lasers were used to excite BODIPY and MitoTracker Red. A Multiple Bessel Beam Light Sheet Pattern with NA max 0.4, NA min 0.38 was used, which has a 75-μm sheet length. The measured lateral resolution was 330 nm and the *z* resolution was 700 nm. To quantify mitochondrial motility and dynamics, we performed cell segmentation, mitochondria segmentation and mitochondria tracking. Single cells were first cropped using ImageJ and Python scripts for all 60 time points. MitoGraph was used to segment the mitochondria in each cell. Based on the segmented mitochondria skeleton, we used MitoTNT to track mitochondria and perform motility calculations with a published protocol^82^. Mitochondria displaying high motility were used for further fusion and fission dynamic analysis. Mitochondria fusion and fission levels were measured by the number of detected events per 1,000 mitochondria skeleton nodes for each frame and only the highly active events (counts > 3) were used for comparison.

### Lipolysis

#### In vitro

Fully differentiated primary adipocytes in a 24-well plate were serum-starved in lipolysis medium (2% BSA-phenol-red-free DMEM) for 3 h. For insulin treatment, 100 nM insulin was added to cells for 30 min starting at 2.5 h of starvation. After starvation, the medium was replaced with 0.5 ml fresh lipolysis medium with vehicle, 1 μM CL, 100 nM insulin or in combination. The medium was collected after 1 h incubation at 37 °C. Released FFAs and free glycerol levels were measured using 100 μl medium with a NEFA kit (WAKO) and Free Glycerol Reagent (Sigma) according to the manufacturer’s protocol.

#### In vivo

CD-fed mice were used for in vivo lipolysis. For CL-induced lipolysis, ad libitum-fed mice were i.p. injected with PBS or CL (1 mg kg^−1^) for 60 min. Circulating FFAs and free glycerol levels were measured using 2 μl plasma with a NEFA kit (WAKO) and Free Glycerol Reagent (Sigma). For insulin-suppressed lipolysis, overnight-fasted mice were i.p. injected with insulin (0.5 U kg^−1^) for 60 min. Circulating FFAs and free glycerol levels were measured at the indicated conditions.

### Electron microscopy

#### Adipose tissue

Dissected adipose tissue was immediately fixed with 2–3 drops of fixative buffer (2% paraformaldehyde and 2.5% glutaraldehyde in 0.15 M sodium cacodylate buffer, pH 7.4). Fat tissues were gently removed and fixed at RT. After 2 h incubation, tissues were further cut into around 1-mm^3^ cubes and immersed in fixative buffer overnight at 4 °C. Tissue cubes were postfixed in 1% osmium 0.15 M sodium cacodylate (SC) buffer for 1–2 h on ice, followed by five 10-min washes in 0.15 M SC buffer, then rinsed in ddH_2_O on ice. Washed tissues were stained with 2% uranyl acetate for 1–2 h at 4 °C then dehydrated in an ethanol series (50%, 70%, 90%, 100% and 100%, for 10 min each time) and dried in acetone for 15 min at RT. Dried tissues were infiltrated with 50:50% acetone:Durcupan for 1 h or longer at RT then changed to 100% Durcupan overnight. The next day, embedded tissues in Durcupan were placed in a 60 °C oven for 36 to 48 h. Ultrathin sections (60 nm) were cut on a Leica microtome with a Diamond knife and then post-stained with both uranyl acetate and lead. Images were obtained using a Jeol 1400 plus TEM equipped with a Gatan digital camera.

#### Immortalized cells

Fully differentiated cells in a six-well plate were quickly fixed with 2% glutaraldehyde in 0.1 M SC buffer (pH 7.4) at RT for 15 min then incubated at 4 °C for 15 min. Afterwards, cells were scraped down and pelleted by centrifugation. Cell pellets were postfixed in 1% OsO_4_ in 0.1 M SC buffer for 1 h on ice. The cells were stained all at once with 2% uranyl acetate for 1 h on ice, then dehydrated in a graded series of ethanol (50–100%) while remaining on ice. The cells were then subjected to one wash with 100% ethanol and two washes with acetone (10 min each) and embedded with Durcupan. Sections were cut at 60 nm on a Leica UCT ultramicrotome and picked up on 300 mesh copper grids. Sections were post-stained with 2% uranyl acetate for 5 min and Sato’s lead stain for 1 min. Images were obtained using a Jeol 1400 plus TEM equipped with a Gatan digital camera.

### cAMP measurement

To induce cAMP production, fully differentiated primary adipocytes were stimulated with 1 μM CL for 5 min. Cells were then immediately lysed in lysis buffer (0.1 N HCL) and cAMP levels were measured with the Direct cAMP Enzyme Immunoassay kit (Sigma) according to the manufacturer’s protocol.

### Pulldown and co-immunoprecipitation

#### Active RalA pulldown

Fully differentiated primary adipocytes or immortalized adipocytes were serum-starved for 3 h in DMEM and treated with 100 nM insulin, if needed, for the indicated time. After two washes with ice-cold TBS, cells were lysed in RalA buffer (25 mM Tris, 130 mM NaCl, 10 mM MgCl_2_, 10% glycerol, 0.5% NP-40 and EDTA-free protease inhibitor) and lysates were incubated at 4 °C for 15 min. then cleared by centrifugation. Protein concentrations were measured with the DC protein assay (Bio-Rad) and 0.5–1 mg protein was used for incubation at 4 °C with 20 μl GST-^Δ^RalBP1 agarose beads (Millipore) for 45 min or 20 μl Anti-Flag M2 Affinity gel (Sigma) overnight. After incubation, beads were washed three times with RalA buffer and boiled at 65 °C in 2× SDS buffer for 10 min.

#### Pulldown

HEK293T cells cultured in 15-cm dishes were transfected with Flag–RalA^WT^ or GFP–PP2Aa. At 48 h after transfection, cells were washed twice with ice-cold TBS then lysed on ice with 1 ml lysis buffer (25 mM Tris-HCl, 130 mM NaCl, 10 mM MgCl_2_, 10% glycerol, 0.5% NP-40 and EDTA-free protease inhibitor). Cell lysates were rotated for 15 min at 4 °C and cleared by centrifugation for 15 min at 17,000*g* at 4 °C. Flag–RalA^WT^ lysates were incubated with 20 μl Anti-Flag M2 Affinity gel (Sigma) at 4 °C. After 2 h rotation, the empty M2 or Flag–RalA^WT^ beads were washed three times with lysis buffer then incubated with GFP–PP2Aa lysates at 4 °C overnight. The next day, the beads were washed three times with washing buffer (25 mM Tris-HCl, 40 mM NaCl, 30 mM MgCl_2_, 0.5% NP-40 and EDTA-free protease inhibitor) and boiled in 2× SDS buffer at 65 °C for 10 min. For GTPγS and GDP loading to Flag–RalA^WT^ beads, washed beads were rinsed with loading buffer (20 mM Tris, 50 mM NaCl, 1 mM dithiothreitol and 2 mM EDTA) then incubated with 2 mM GTPγS or 200 μM GDP in loading buffer for 1 h at 25 °C with 50*g* agitation. After loading, 10 mM MgCl_2_ was added to stop the loading and loaded beads were incubated with GFP–PP2Aa lysates as described above.

#### Co-immunoprecipitation

Co-transfected cells at 70–80% confluency were washed twice with ice-cold TBS and lysed in 0.5 ml lysis buffer or Drp1 buffer (25 mM Tris, 50 mM NaCl, 0.5 mM MgCl_2_, 10% glycerol, 0.5% NP-40 and EDTA-free protease inhibitor). Lysates were cleared by centrifugation and protein concentrations were measured with BCA (Pierce). Then, 0.5–1 mg protein was used for incubation with 20 μl Anti-Flag M2 Affinity gel (Sigma) at 4 °C. After overnight gentle rotation, beads were washed three times with washing buffer (the same as described above) or Drp1 wash buffer (25 mM Tris, 50 mM NaCl, 0.5 mM MgCl_2_, 0.1% NP-40 and EDTA-free protease inhibitor) and boiled in 2× SDS buffer at 65 °C for 10 min.

### Vector construction

pMIG-PP2Aa (#10884), pMIG-PP2Ab (#13804) and pcDNA3.1-Drp1 (#34706) plasmids were purchased from Addgene and subcloned into mEGFP-C1 (#54759) and pCMV-Myc-3B vectors. RalA^WT^, RalA^G23V^ and RalA^S28N^ plasmids were subcloned into a pLVX vector with 3× Flag tag for lentiviral production.

### Statistics and reproducibility

All in vivo animal experiments were randomized by genotype and the investigators were not blinded to allocation during experiments and outcome assessment. All in vitro cell experiments were not randomized. There was no predetermination of sample size and sample size was chosen based on available animal or cell numbers. Negative values or Prism-detected outliners were excluded from the analyses due to poor sample quality or samples lost during processing. Statistical analyses were performed using GraphPad Prism (v.8.4.3). All experiments were performed at least three times independently. Data distribution was assumed to be normal without formal testing. For comparison between two groups, datasets were analyzed by a two-tailed Student’s *t*-test. For experiments with a two-factorial design, multiple comparisons were analyzed by two-way ANOVA to determine the statistical significance between groups based on one variable. Differences in EE were calculated with CalR using ANCOVA with BW as a covariate. The significance of the correlations between gene expression with BMI and HOMA values were calculated using a Spearman’s correlation test. Values of *P* < 0.05 were considered as significant.

### Schematics

Schematic graphs were created with Biorender.com.

### Reporting summary

Further information on research design is available in the Nature Portfolio Reporting Summary linked to this article.

### Supplementary information

Reporting Summary

Supplementary Video 1 Mitochondrial 3D imaging video in KO cells.

Supplementary Video 2 Mitochondrial 3D imaging video in KO+RalA^WT^ cells.

### Source data

Source Data Fig. 1 Statistical source data.

Source Data Fig. 2 Statistical source data.

Source Data Fig. 3 Statistical source data.

Source Data Fig. 3 Uncropped western blots.

Source Data Fig. 4 Statistical source data.

Source Data Fig. 5 Statistical source data.

Source Data Fig. 5 Uncropped western blots.

Source Data Fig. 6 Statistical source data.

Source Data Fig. 6 Uncropped western blots.

Source Data Extended Data Fig. 1 Statistical source data.

Source Data Extended Data Fig. 1 Uncropped western blots.

Source Data Extended Data Fig. 2 Statistical source data.

Source Data Extended Data Fig. 3 Statistical source data.

Source Data Extended Data Fig. 3 Uncropped western blots.

Source Data Extended Data Fig. 4 Statistical source data.

Source Data Extended Data Fig. 4 Uncropped western blots.

Source Data Extended Data Fig. 5 Statistical source data.

Source Data Extended Data Fig. 5 Uncropped western blots.

Source Data Extended Data Fig. 6 Statistical source data.

Source Data Extended Data Fig. 6 Uncropped western blots.

Source Data Extended Data Fig. 7 Statistical source data.

Source Data Extended Data Fig. 7 Uncropped western blots.

## Figures and Tables

**Extended Data Table 1:**
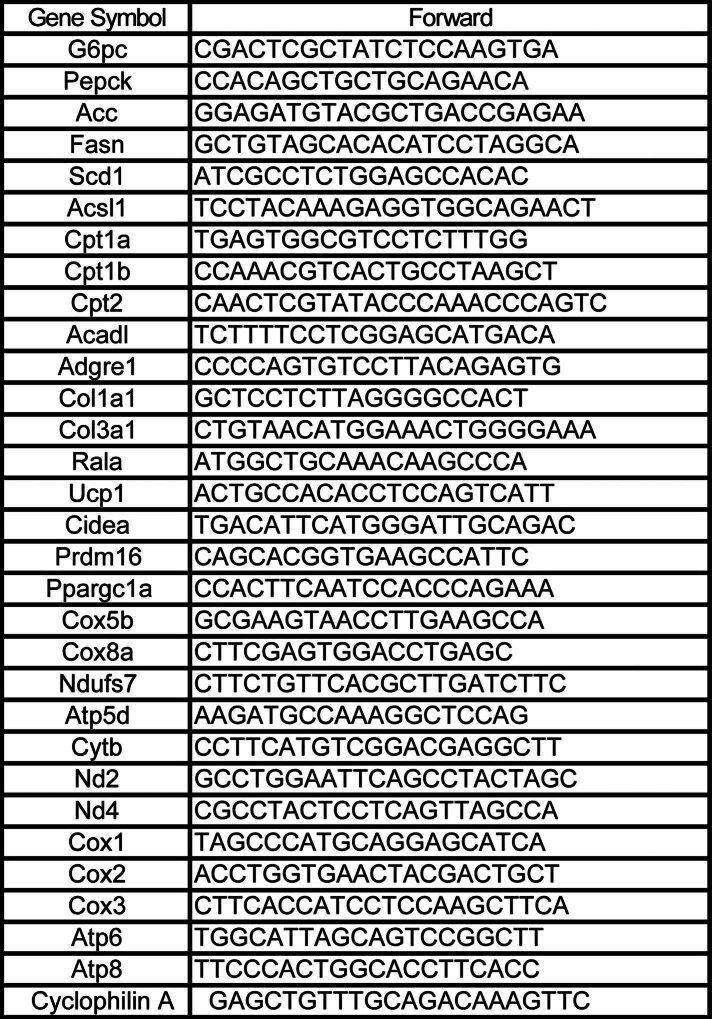
Real-time qPCR primers

qPCR primers

## Data Availability

All data supporting the findings of this study are available within the paper and its Supplementary Information. Source data and uncropped western blot gels are provided with this paper. qPCR primer sequences are provided in Extended Data Table 1. RNA-seq data reported in this paper have been deposited in the NCBI SRA database (BioProject PRJNA727566). Human study data are deposited in the NCBI GEO under accession code GSE25402 and retrieved from GEO (GSE70353). Genome sequences were from genome mm39 (PRJNA20689). Source data are provided with this paper.

## Code availability

Adipocyte size from CD-fed mice was assayed using CellProfiler with an in-house-modified pipeline available at https://github.com/Wenmin2023/Adipocyte-quantification-pipeline.
