# Supplementary material for: Obesity causes mitochondrial fragmentation and dysfunction in white adipocytes due to RalA activation
Source: Nat Metab. 2024 Jan 29;6(2):273–89. doi: 10.1038/s42255-024-00978-0 (PMC10896723; doi:10.1038/s42255-024-00978-0)
Supplement: Supplementary file 1 — Reporting Summary [file 42255_2024_978_MOESM1_ESM.pdf]

nature portfolio

Corresponding author(s): Alan Saltiel

Last updated by author(s): Dec 11, 2023

Reporting Summary

Nature Portfolio wishes to improve the reproducibility of the work that we publish. This form provides structure for consistency and transparency in reporting. For further information on Nature Portfolio policies, see our Editorial Policies and the Editorial Policy Checklist.

Statistics

For all statistical analyses, confirm that the following items are present in the figure legend, table legend, main text, or Methods section.

n/a	Confirmed	
☐	☒	The exact sample size (*n*) for each experimental group/condition, given as a discrete number and unit of measurement
☐	☒	A statement on whether measurements were taken from distinct samples or whether the same sample was measured repeatedly
☐	☒	The statistical test(s) used AND whether they are one- or two-sided<br>*Only common tests should be described solely by name; describe more complex techniques in the Methods section.*
☐	☒	A description of all covariates tested
☐	☒	A description of any assumptions or corrections, such as tests of normality and adjustment for multiple comparisons
☐	☒	A full description of the statistical parameters including central tendency (e.g. means) or other basic estimates (e.g. regression coefficient) AND variation (e.g. standard deviation) or associated estimates of uncertainty (e.g. confidence intervals)
☒	☐	For null hypothesis testing, the test statistic (e.g. *F*, *t*, *r*) with confidence intervals, effect sizes, degrees of freedom and *P* value noted<br>*Give P values as exact values whenever suitable.*
☒	☐	For Bayesian analysis, information on the choice of priors and Markov chain Monte Carlo settings
☒	☐	For hierarchical and complex designs, identification of the appropriate level for tests and full reporting of outcomes
☒	☐	Estimates of effect sizes (e.g. Cohen's *d*, Pearson's *r*), indicating how they were calculated

Our web collection on statistics for biologists contains articles on many of the points above.

Software and code

Policy information about availability of computer code

Data collection	EchoMRI v2018, Keyance BZ-X700, iTecan v2.0, Wave Controller for XEf v2.4, NIS-Elements v4.51.01, ExpeData v1.9.27, QuantStudio v1.5.1, NanoDrop2000 v1.3.1, SerialTEM 3.1.1a, Illumina bcl2fastq2 Conversion Software v2.20, LI-COR Odyssey XF Imager, Agilent Seahorse Wave 2.6.1.
Data analysis	Microsoft® Excel® for Microsoft 365 MSO (Version 2304 Build 16.0.16327.20200) 64-bit, Fiji ImageJ(v1.52p), Cell Profiler(3.1.5), GraphPad Prism(8.4.3), CalR(v1.3), STAR (version 2.7.2b), GENE-E (v3.0.215), GSEA (4.3.2), ComparativeMarkerSelection module (version 11), Image Studio Lite(V5.2).

For manuscripts utilizing custom algorithms or software that are central to the research but not yet described in published literature, software must be made available to editors and reviewers. We strongly encourage code deposition in a community repository (e.g. GitHub). See the Nature Portfolio guidelines for submitting code & software for further information.

Data

Policy information about availability of data

All manuscripts must include a data availability statement. This statement should provide the following information, where applicable:

- Accession codes, unique identifiers, or web links for publicly available datasets
- A description of any restrictions on data availability
- For clinical datasets or third party data, please ensure that the statement adheres to our policy

RNA-Seq data reported in this paper have been deposited in NCBI SRA database (BioProject PRJNA727566), human data are deriving from published database GSE25402 and GSE70353, mouse genome sequence information is from BioProject PRJNA20689.

Research involving human participants, their data, or biological material

Policy information about studies with human participants or human data. See also policy information about sex, gender (identity/presentation), and sexual orientation and race, ethnicity and racism.

Reporting on sex and gender	All the information about human participants has been previously published and referenced in the manuscript.
Reporting on race, ethnicity, or other socially relevant groupings	All the information about human participants has been previously published and referenced in the manuscript.
Population characteristics	All the information about human participants has been previously published and referenced in the manuscript.
Recruitment	All the information about human participants has been previously published and referenced in the manuscript.
Ethics oversight	All the information about human participants has been previously published and referenced in the manuscript.

Note that full information on the approval of the study protocol must also be provided in the manuscript.

Field-specific reporting

Please select the one below that is the best fit for your research. If you are not sure, read the appropriate sections before making your selection.

☒ Life sciences ☐ Behavioural & social sciences ☐ Ecological, evolutionary & environmental sciences

For a reference copy of the document with all sections, see nature.com/documents/nr-reporting-summary-flat.pdf

Life sciences study design

All studies must disclose on these points even when the disclosure is negative.

Sample size	No predetermination of sample size was performed for animal experiments. Sample size was determined by the available number of age-matched litters. Sample size (n) for each experiment is included in the figure legend. Experiments were performed with at least 2 replicates.
Data exclusions	No data were excluded from analyses.
Replication	For primary cell culture, three independent isolations were performed and similar results were observed. For immortalized cell, three different single cell clone were used and similar results were observed within those clones. Experiments in which statistical analysis was not applicate were repeated at least three times, other experiments were repeated at least twice.
Randomization	For in vivo study, injection order to mice were assigned based on their housing cage and ear tag. For metabolic study, two genotypes were crossly assigned to avoid confounding by cage location effect.
Blinding	The researcher was blinded to the genotype during data collection, this includes blinded process of EM sample. During data analysis, the researchers were not blinded to allocation since data comparison needs to be done between different genotypes or groups.

Reporting for specific materials, systems and methods

We require information from authors about some types of materials, experimental systems and methods used in many studies. Here, indicate whether each material, system or method listed is relevant to your study. If you are not sure if a list item applies to your research, read the appropriate section before selecting a response.

Materials & experimental systems

n/a	Involved in the study	
☐	☒	Antibodies
☐	☒	Eukaryotic cell lines
☒	☐	Palaeontology and archaeology
☐	☒	Animals and other organisms
☒	☐	Clinical data
☒	☐	Dual use research of concern
☒	☐	Plants

Methods

n/a	Involved in the study	
☒	☐	ChIP-seq
☒	☐	Flow cytometry
☒	☐	MRI-based neuroimaging

Antibodies

Antibodies used

OXPHOS (Abcam, ab110413), β-Tubulin (CST 2146S), phospho-Drp1(Ser637) (CST 4867S), phospho-HSL(Ser660) (CST 45804S), HSL (CST 4107S), MYC (CST 2276S), Drp1 (CST 8570S), phosphor-AMPK(Thr172) (CST 2535S), AMPK (CST 5831S), RalA (BD Science BD610221), β-Actin (ProteinTech 66009-1-Ig), FLAG (ProteinTech 66008-4-Ig), GFP (ProteinTech 66002-1-Ig), Sec5 (ProteinTech 12751-1-AP), GLUT4 (Millipore 07-1404), IRAP (CST 6918S), Na,K-ATPase (CST 3010S). All primary antibodies for WB was diluted as 1:1000.

Validation

All antibodies were validated by manufacturer and confirmed with western blot by their expected change upon treatment or in KO cells.
OXPHOS: https://www.abcam.com/products/panels/total-oxphos-rodent-wb-antibody-cocktail-ab110413.html
β-Tubulin: https://www.cellsignal.com/products/primary-antibodies/b-tubulin-antibody/2146?site-search-type=Products&N=4294956287&Ntt=2146s&fromPage=plp&_requestid=807705
phospho-Drp1(Ser637): https://www.cellsignal.com/products/primary-antibodies/phospho-drp1-ser637-antibody/4867?site-search-type=Products&N=4294956287&Ntt=4867s&fromPage=plp&_requestid=795386
phospho-HSL(Ser660): https://www.cellsignal.com/products/primary-antibodies/phospho-hsl-ser660-antibody/45804?site-search-type=Products&N=4294956287&Ntt=45804s&fromPage=plp&_requestid=795885
HSL: https://www.cellsignal.com/products/primary-antibodies/hsl-antibody/4107?site-search-type=Products&N=4294956287&Ntt=4107s&fromPage=plp&_requestid=796018
MYC: https://www.cellsignal.com/products/primary-antibodies/myc-tag-9b11-mouse-mab/2276?site-search-type=Products&N=4294956287&Ntt=2276s&fromPage=plp&_requestid=796144
Drp1: https://www.cellsignal.com/products/primary-antibodies/drp1-d6c7-rabbit-mab/8570?site-search-type=Products&N=4294956287&Ntt=8570s&fromPage=plp&_requestid=796353
phospho-AMPK(Thr172): https://www.cellsignal.com/products/primary-antibodies/phospho-ampka-thr172-40h9-rabbit-mab/2535?site-search-type=Products&N=4294956287&Ntt=2535s&fromPage=plp&_requestid=797106
AMPK: https://www.cellsignal.com/products/primary-antibodies/ampka-d5a2-rabbit-mab/5831?site-search-type=Products&N=4294956287&Ntt=5831s&fromPage=plp&_requestid=797159
RalA: https://www.bdbiosciences.com/en-eu/products/reagents/microscopy-imaging-reagents/immunofluorescence-reagents/purified-mouse-anti-ral-a.610221
β-Actin: https://www.ptglab.com/products/Pan-Actin-Antibody-66009-1-Ig.htm
FLAG: https://www.ptglab.com/products/Flag-tag-Antibody-66008-4-Ig.htm
GFP: https://www.ptglab.com/products/eGFP-Antibody-66002-1-Ig.htm
Sec5: https://www.ptglab.com/products/SEC5-Antibody-12751-1-AP.htm
GLUT4: https://www.emdmillipore.com/US/en/product/Anti-GLUT-4-Antibody-C-terminus,MM_NF-07-1404
IRAP: https://www.cellsignal.com/products/primary-antibodies/irap-d7c5-xp-rabbit-mab/6918
Na,K-ATPase: https://www.cellsignal.com/products/primary-antibodies/na-k-atpase-antibody/3010

Eukaryotic cell lines

Policy information about cell lines and Sex and Gender in Research

Cell line source(s)

3T3-L1 and HEK293T cells were purchased from American Type Culture Collection. LentiX 293T cells were purchased from Takara. Primary cells were freshly isolated in lab and immediately used for experiment. Immortalized primary cells were generated in house, they are named as WT, KO, KO+RalAWT and KO+RalAG23V in the manuscript.

Authentication

Commercial cell lines were validated by American Type Culture Collection, primary and immortalized cells were validated in the lab (preadipocytes: efficient adipogenesis, LentiX 293: efficient virus production). Those cells were authenticated mainly by their morphology.

Mycoplasma contamination

Cell lines were not tested for mycoplasma contamination.

Commonly misidentified lines
(See ICLAC register)

N/A.

Animals and other research organisms

Policy information about studies involving animals; ARRIVE guidelines recommended for reporting animal research, and Sex and Gender in Research

Laboratory animals	2-4 months old male mouse with C57BL/6 background were used for in vivo study, 8 weeks old female mouse with C57BL/6 were only used for primary cell isolation.
Wild animals	The study did not involve wild animals.
Reporting on sex	Physiological and pathological findings in this study only apply to male mice. For C57BL6/J genetic background, only male mice can be induced to metabolic dysfunction with high fat diet feeding.
Field-collected samples	The study did not involve samples collected from field.
Ethics oversight	Institutional Animal Care and Use Committee (IACUC) at the University of California San Diego approved and provide guidance on the study protocol.

Note that full information on the approval of the study protocol must also be provided in the manuscript.
