# Supplementary figures and images for: Obesity causes mitochondrial fragmentation and dysfunction in white adipocytes due to RalA activation
Source: Nat Metab. 2024 Jan 29;6(2):273–89. doi: 10.1038/s42255-024-00978-0 (PMC10896723; doi:10.1038/s42255-024-00978-0)

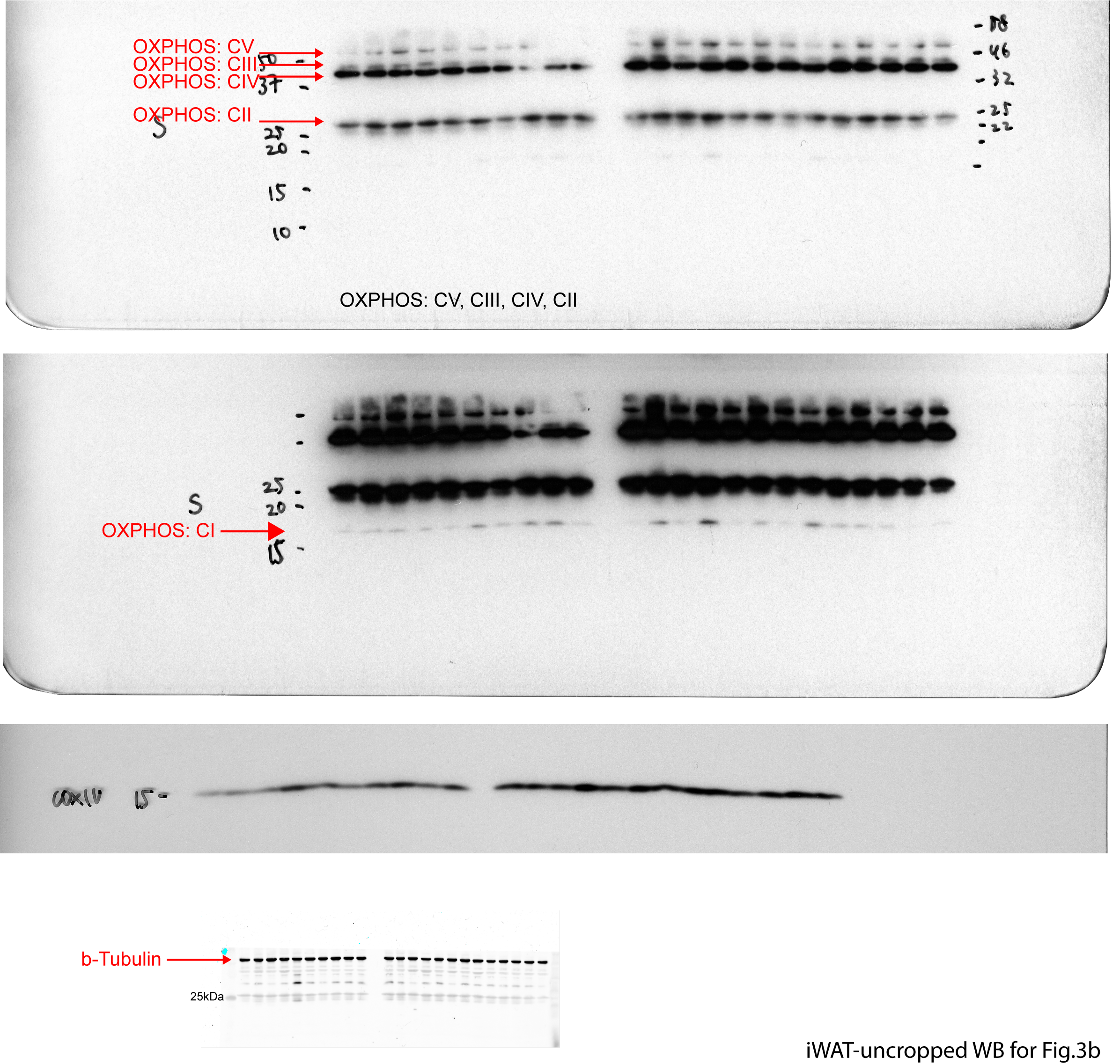

Supplement: Supplementary file 7 — Uncropped western blots. [file 42255_2024_978_MOESM7_ESM.tif]

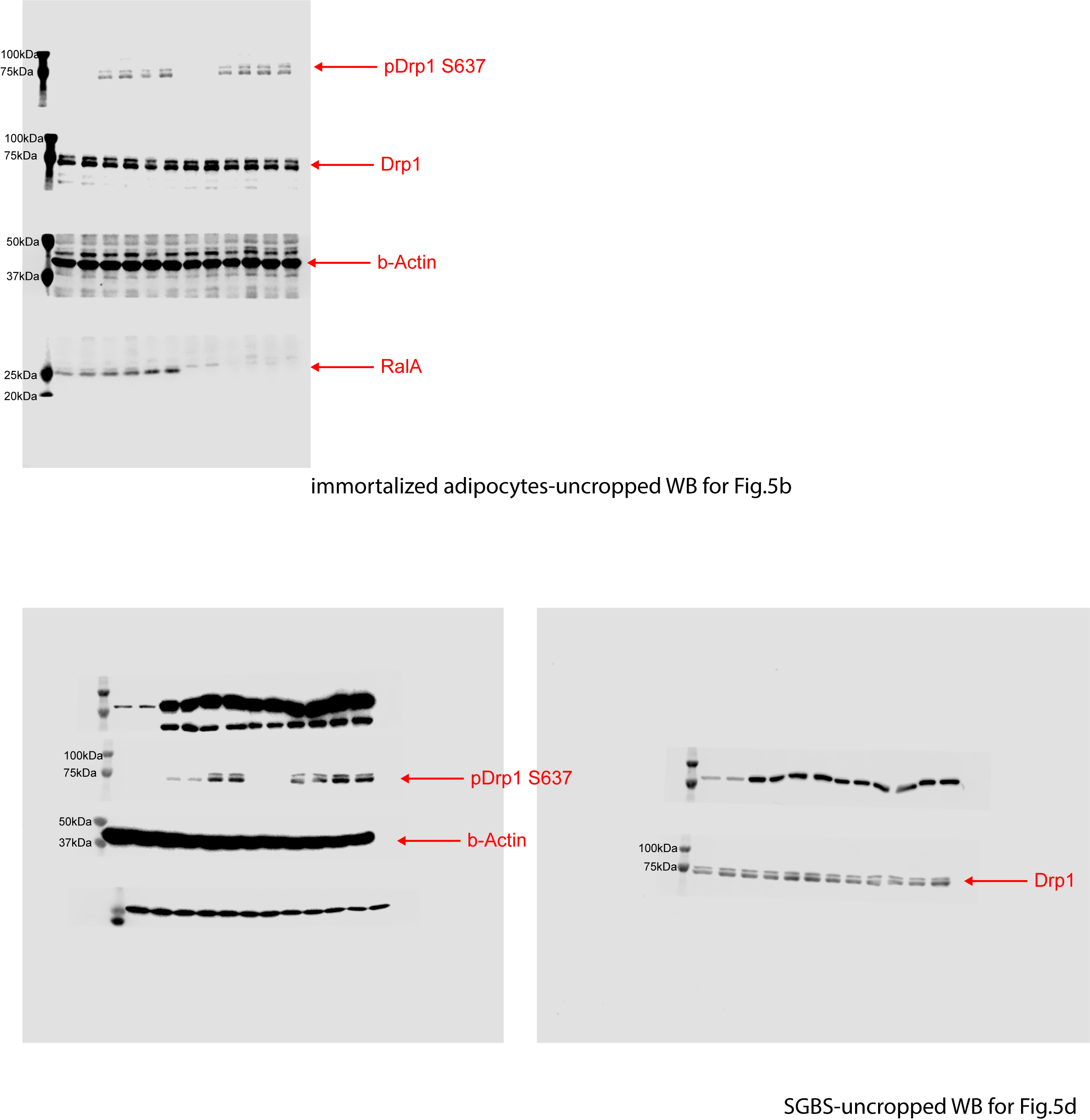

Supplement: Supplementary file 10 — Uncropped western blots. [file 42255_2024_978_MOESM10_ESM.tif]

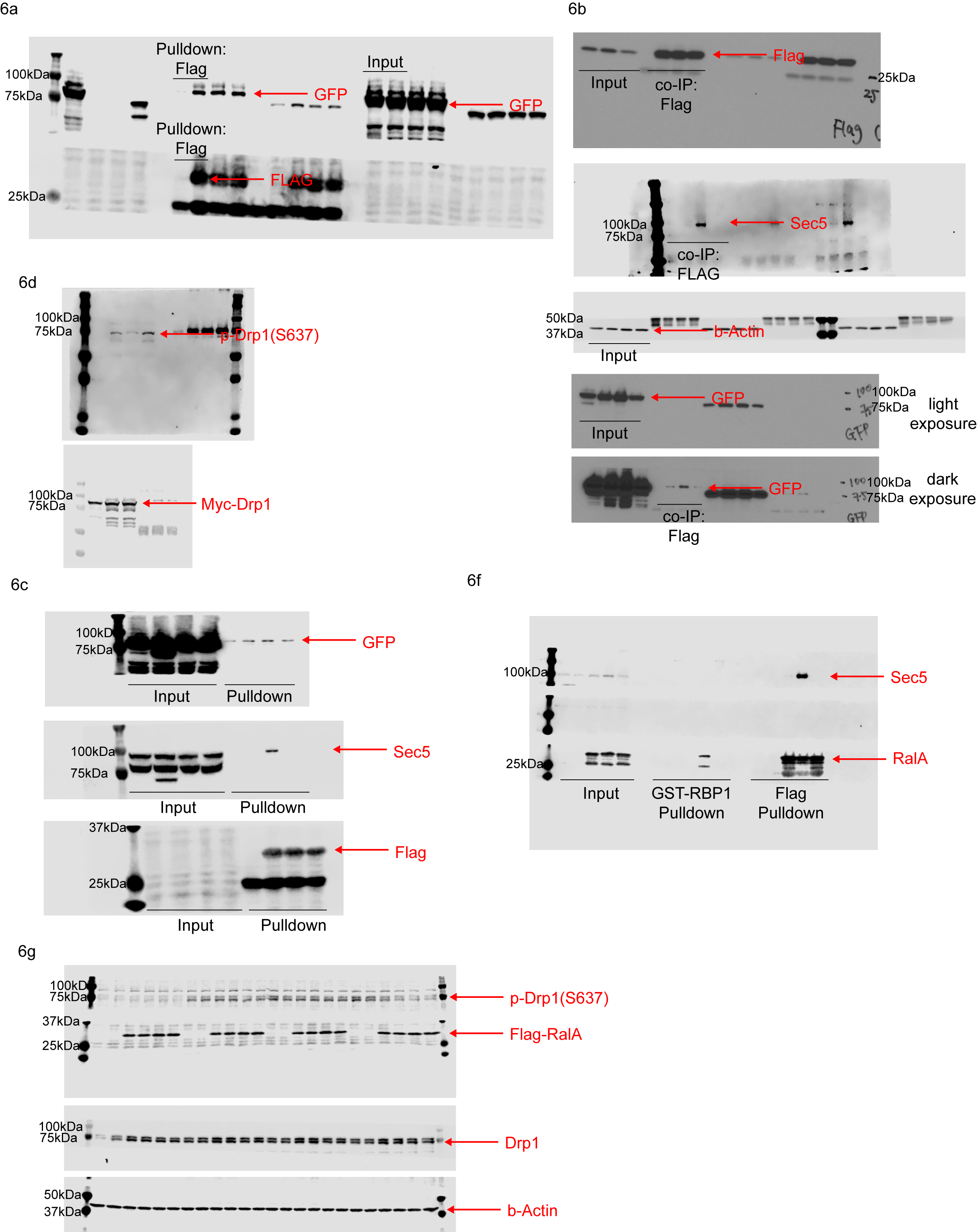

Supplement: Supplementary file 12 — Uncropped western blots. [file 42255_2024_978_MOESM12_ESM.tif]

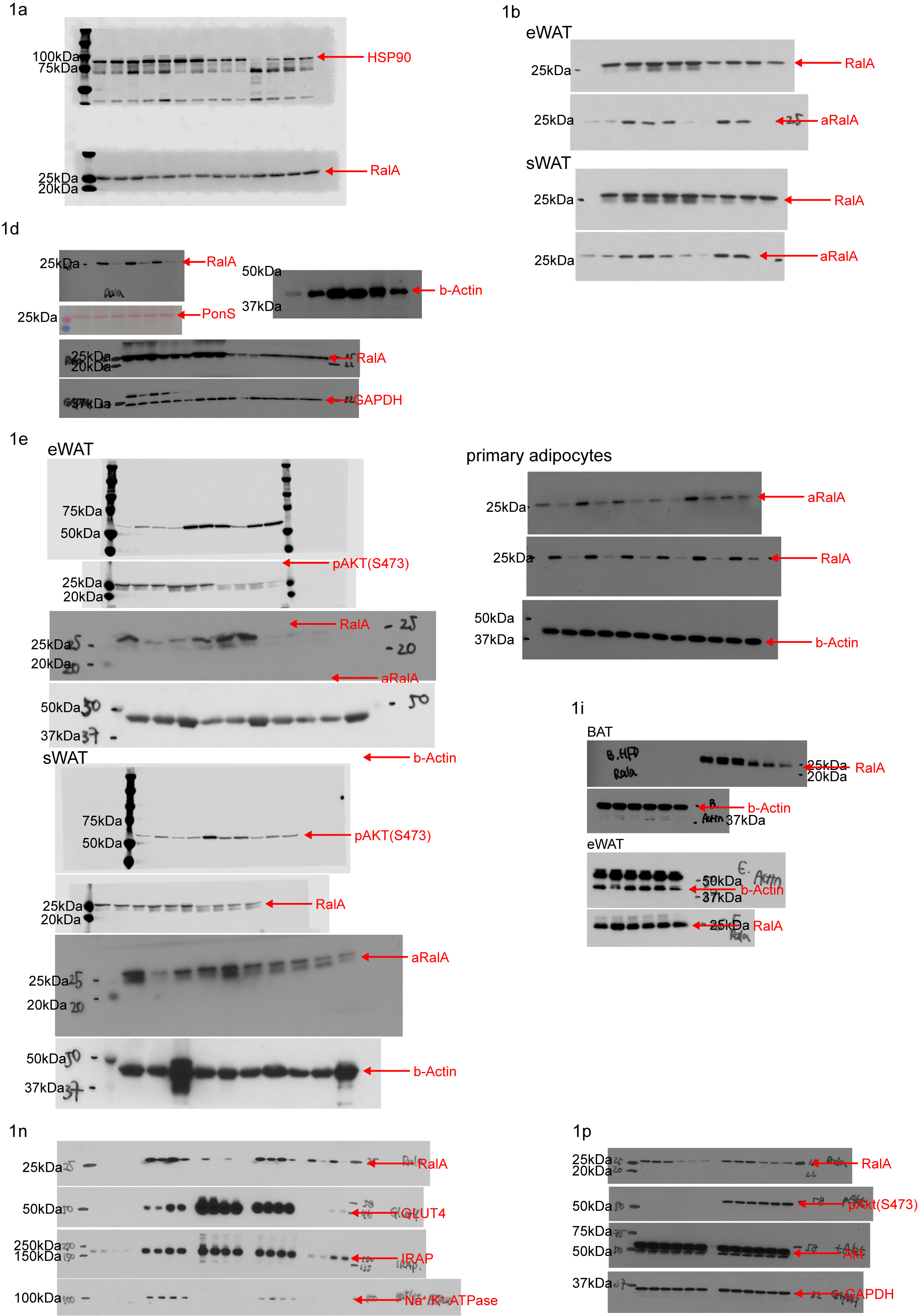

Supplement: Supplementary file 14 — Uncropped western blots. [file 42255_2024_978_MOESM14_ESM.tif]

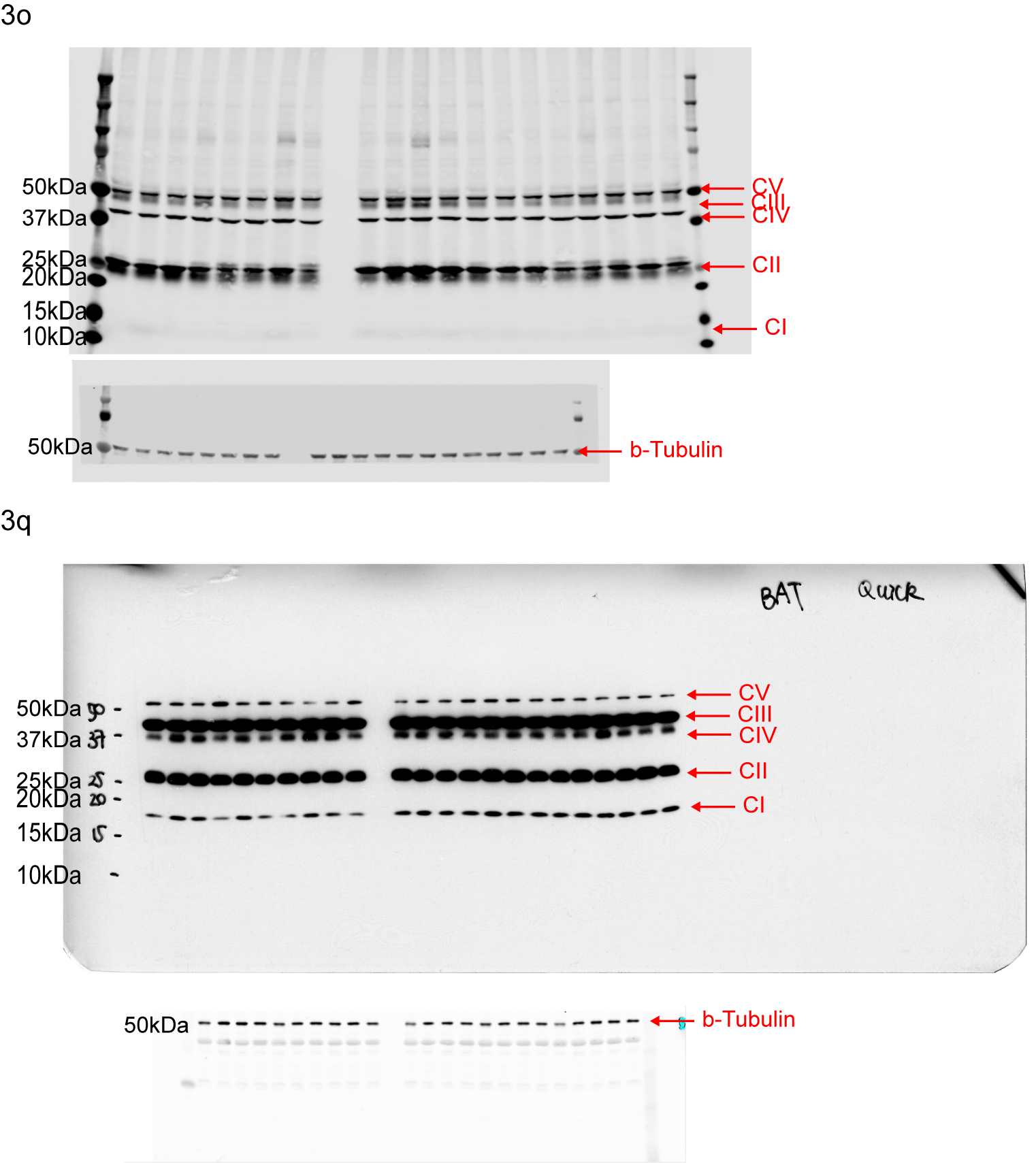

Supplement: Supplementary file 17 — Uncropped western blots. [file 42255_2024_978_MOESM17_ESM.tif]

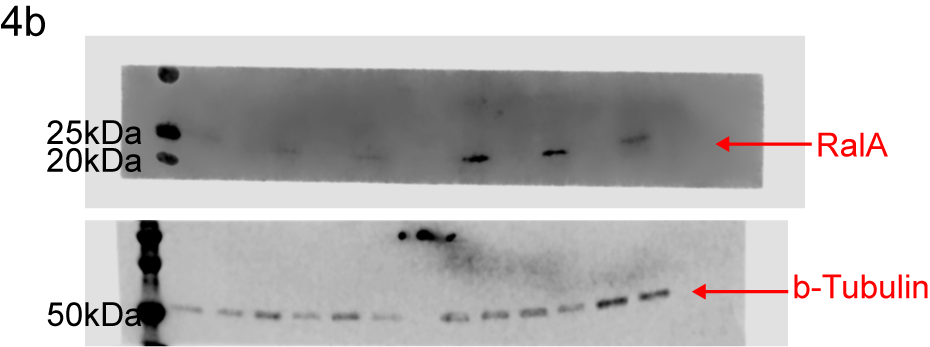

Supplement: Supplementary file 19 — Uncropped western blots. [file 42255_2024_978_MOESM19_ESM.tif]

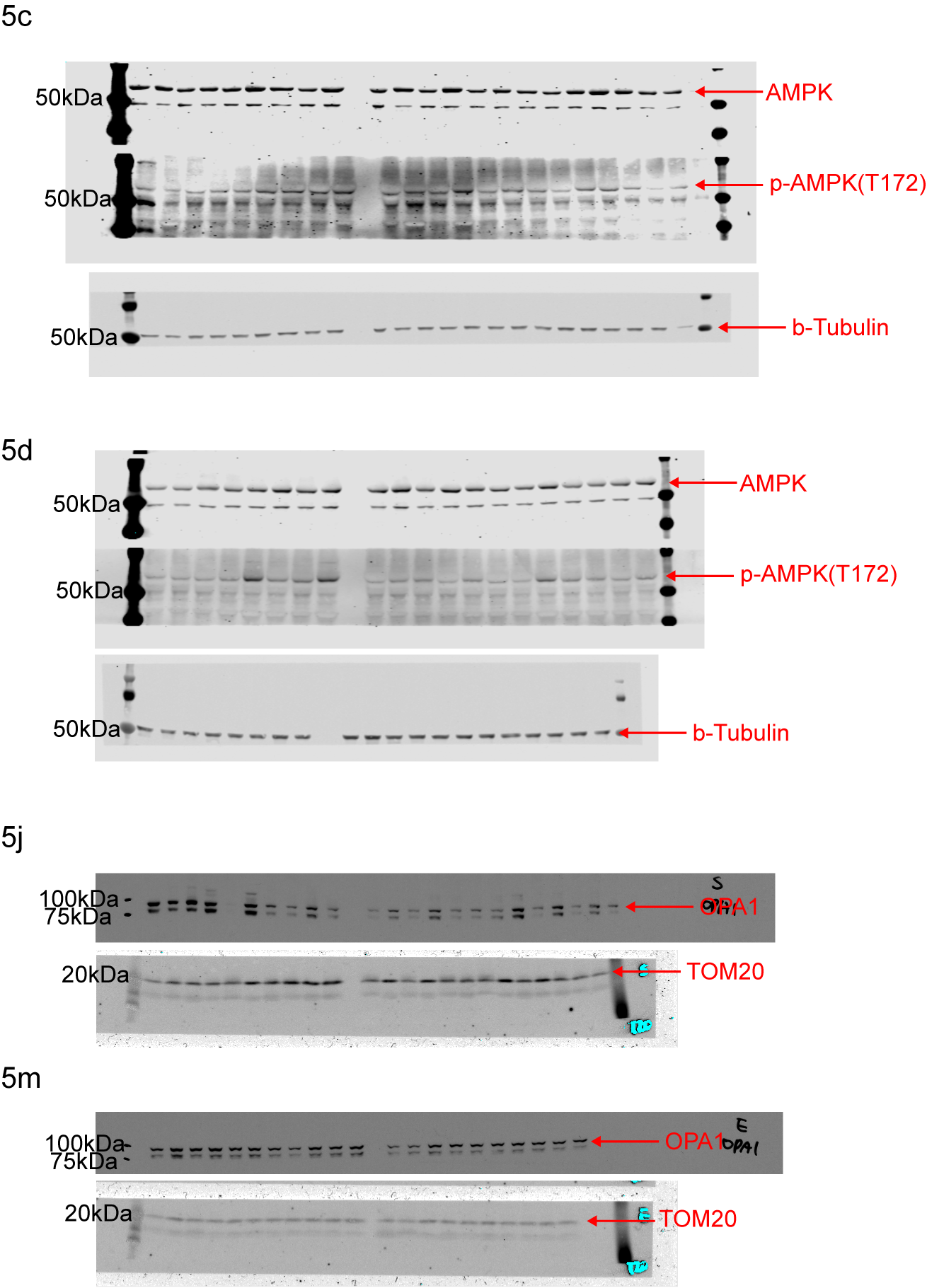

Supplement: Supplementary file 21 — Uncropped western blots. [file 42255_2024_978_MOESM21_ESM.tif]

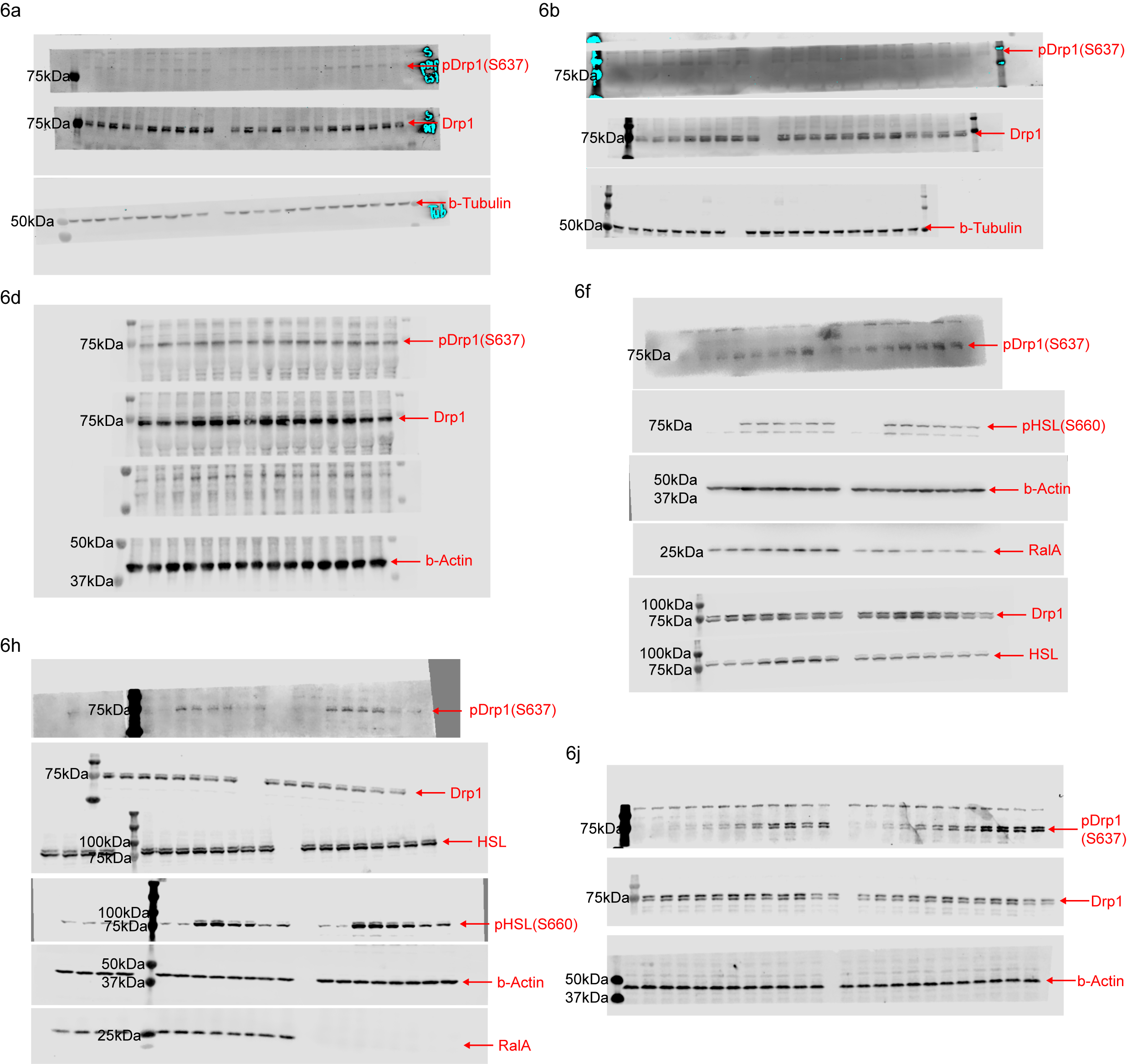

Supplement: Supplementary file 23 — Uncropped western blots. [file 42255_2024_978_MOESM23_ESM.tif]

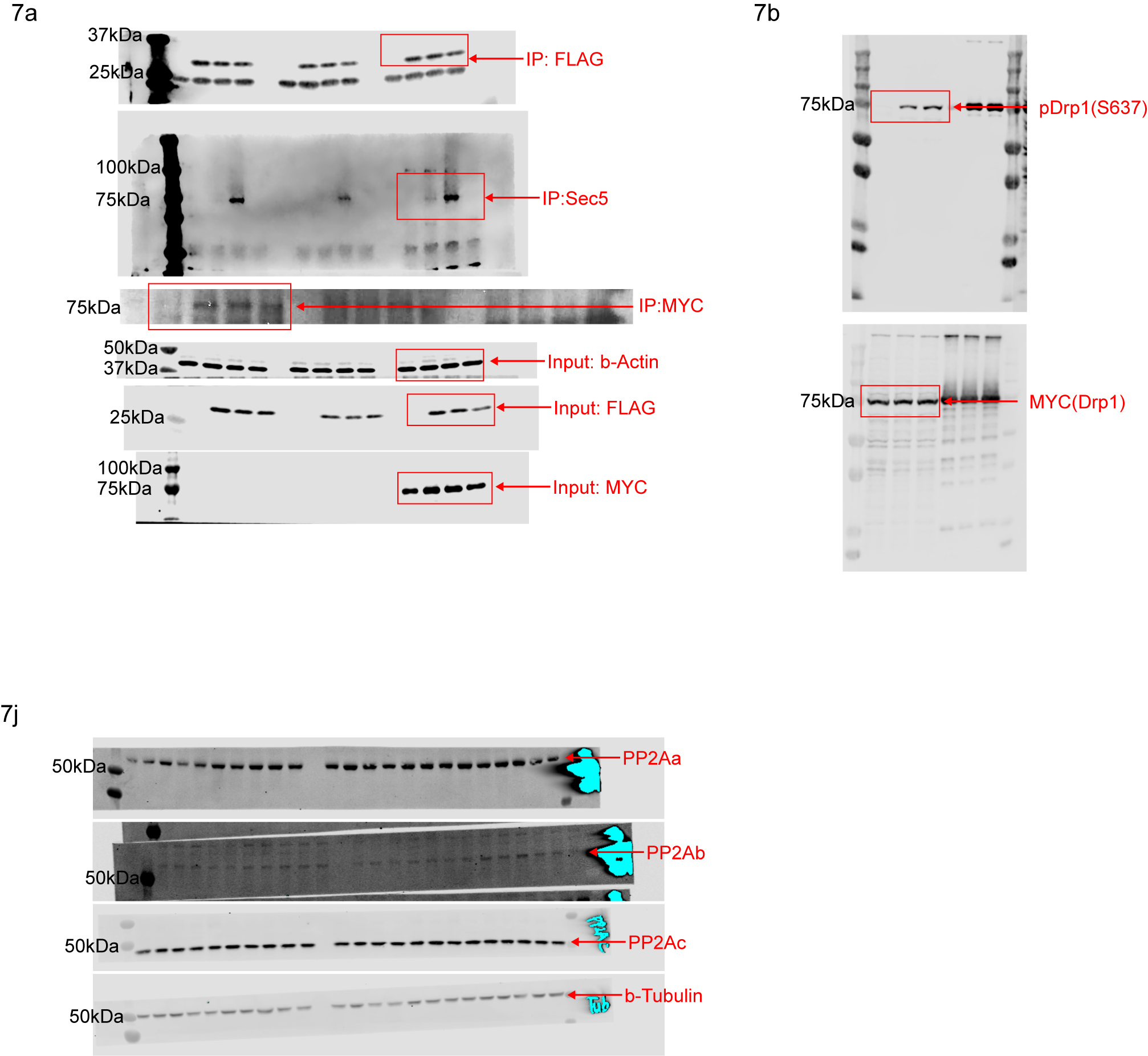

Supplement: Supplementary file 25 — Uncropped western blots. [file 42255_2024_978_MOESM25_ESM.tif]
